# Globally distributed mining-impacted environments are underexplored hotspots of multidrug resistance genes

**DOI:** 10.1038/s41396-022-01258-z

**Published:** 2022-06-10

**Authors:** Xinzhu Yi, Jie-Liang Liang, Jian-Qiang Su, Pu Jia, Jing-li Lu, Jin Zheng, Zhang Wang, Shi-wei Feng, Zhen-hao Luo, Hong-xia Ai, Bin Liao, Wen-sheng Shu, Jin-tian Li, Yong-Guan Zhu

**Affiliations:** 1 Institute of Ecological Science, Guangzhou Key Laboratory of Subtropical Biodiversity and Biomonitoring, Guangdong Provincial Key Laboratory of Biotechnology for Plant Development, School of Life Sciences, South China Normal University, Guangzhou, 510631 PR China; 2 Key Lab of Urban Environment and Health, Institute of Urban Environment, Chinese Academy of Sciences, Xiamen, 361021 PR China; 3 School of Life Sciences, Sun Yat-sen University, Guangzhou, 510275 PR China; 4 Guangdong Provincial Key Laboratory of Chemical Pollution, South China Normal University, Guangzhou, 510006 PR China

**Keywords:** Microbial ecology, Environmental microbiology

## Abstract

Mining is among the human activities with widest environmental impacts, and mining-impacted environments are characterized by high levels of metals that can co-select for antibiotic resistance genes (ARGs) in microorganisms. However, ARGs in mining-impacted environments are still poorly understood. Here, we conducted a comprehensive study of ARGs in such environments worldwide, taking advantage of 272 metagenomes generated from a global-scale data collection and two national sampling efforts in China. The average total abundance of the ARGs in globally distributed studied mine sites was 1572 times per gigabase, being rivaling that of urban sewage but much higher than that of freshwater sediments. Multidrug resistance genes accounted for 40% of the total ARG abundance, tended to co-occur with multimetal resistance genes, and were highly mobile (e.g. on average 16% occurring on plasmids). Among the 1848 high-quality metagenome-assembled genomes (MAGs), 85% carried at least one multidrug resistance gene plus one multimetal resistance gene. These high-quality ARG-carrying MAGs considerably expanded the phylogenetic diversity of ARG hosts, providing the first representatives of ARG-carrying MAGs for the Archaea domain and three bacterial phyla. Moreover, 54 high-quality ARG-carrying MAGs were identified as potential pathogens. Our findings suggest that mining-impacted environments worldwide are underexplored hotspots of multidrug resistance genes.

## Introduction

Antibiotic resistance is one of the biggest global public health threats facing humanity [1]. Antibiotic-resistant infections currently kill approximately 700,000 people per year around the world and are predicted to cause 10,000,000 deaths per year by 2050 [2, 3]. The emergence of antibiotic resistance genes (ARGs) in microorganisms predates human use of antibiotics [4, 5]. However, it cannot be disputed that human activities dramatically accelerate the proliferation and transmission of ARGs [4]. Apart from antibiotics, many other environmental pollutants (particularly metals) are increasingly recognized as important selective agents to promote the dissemination of ARGs in the environment [6, 7].

Among the reported selective agents for ARGs, metals differ greatly from their organic counterparts due to their persistent nature and higher potential to accumulate to selecting levels [6]. As such, metals can be an even more important risk factor for the proliferation and transmission of ARGs in the environment than other selective agents [6, 7]. In the past years, the potential correlations between metal level and ARG abundance or diversity in a variety of environments impacted by agriculture [8], animal husbandry, aquaculture [9, 10], urbanization [11, 12] and oil spill [13] have been studied extensively. However, these focal environments are not exempt from the influences of other selective agents, which has heavily hampered a comprehensive assessment of the direct roles of metals in ARGs proliferation and dissemination in the environment [6, 7].

Mining is one of the major human activities with widest environmental impacts [14], and mining-impacted environments characterized by high levels of metals are ideal settings to study the direct effects of metals on environmental ARGs [6]. However, very little is currently known about the ARGs in mining-impacted environments, especially their linkages with metals or metal resistance genes (MRGs), and their mobility, biogeography as well as hosts [15]. Unlike other human activities such as agriculture and animal husbandry, there is no demand for the use of antibiotics in mining practices [14]. In this context, mining-impacted environments generally are unlikely to be polluted by anthropogenic antibiotics. Typical hazardous metal-rich wastes generated by mining activities consist mainly of mine tailings and acid mine drainage (AMD) [16, 17]. Open dumping is a main disposal route for mine tailings around the world and it has been estimated that over 700,000 tons of metals in mine tailings are disposed on land per year globally [17]. When exposed to air and water, sulfur-bearing mine tailings (and other solid mine wastes) in disposal sites are readily acidified by iron- and sulfur-oxidizing microorganisms to generate a huge quantity of AMD [16]. Under acidic conditions, even though some antibiotics can be produced naturally by microorganisms in mining-impacted environments, they will degrade rapidly [18]. Therefore, we hypothesize that metal pollution is the main cause of ARG dissemination in mining-impacted environments (i.e. hypothesis 1) and that the profiles of ARGs in such environments likely differ from those in environments polluted by antibiotics (hypothesis 2). Given that many reported metal-induced ARGs are commonly located together with MRGs on the same plasmid or mobile genetic element (i.e. a co-selection mechanism termed as “co-resistance”) [6], we also hypothesize that the ARGs in mining-impacted environments are closely related to MRGs and highly mobile (hypothesis 3). Because the profiles of metal-induced ARGs in the environment may differ considerably across study sites with different soil types [19], our fourth hypothesis predicts that at large spatial scales the ARG profiles of mining-impacted environments exhibit apparent geographical distribution patterns. Finally, we hypothesize that, due to the distinct differences in microbial community composition between mining-impacted and antibiotic-polluted environments [20, 21], some previously unrecognized ARG hosts exist in mining-impacted environments (hypothesis 5).

To test our hypotheses, we first conducted a scoping review, and then employed 272 metagenomes obtained from a global-scale data collection and two national sampling efforts in China to characterize ARGs in 75 mine sites distributed globally. We focused our analyses not only on the abundance, diversity, composition, and potential causes of ARGs in the studied mine sites but also on their relationship with MRGs, and their mobility, biogeography as well as hosts. Additionally, a direct comparison of mine sites and antibiotic-polluted environments in ARG profile was made with 30 mine waste metagenomes generated from our national sampling efforts and 60 public metagenomes downloaded from the Sequence Read Archive (SRA) database (including 30 untreated urban sewage metagenomes as well as 30 freshwater sediment metagenomes). As a whole, the results presented here significantly improve our understanding of ARGs in globally distributed mine sites, indicating that the potential risks associated with the ARGs in mining-impacted environments worldwide deserve more attention than they have received in the global ARGs research to date.

## Materials and methods

### A comprehensive literature search and analysis

Exploring ARGs in mining-impacted environments is an emerging area of interest in the ARGs research field. In order to evaluate the current state of knowledge in this emerging area, we conducted a scoping review according to the PRISMA extension for scoping reviews [22]. On 21 September 2021, we searched ISI Web of Science Core Collection for studies aimed to address ARGs in mining-impacted environments, using “antibiotic resistance genes” AND “mining environment” as the topic fields. The database coverage was 1985 to present. We retrieved 67 records published from 1991 to 2021. To be included in our review, we required that the study explicitly addressed microbial ARGs or antibiotic resistances (ARs) in mining-impacted environments. Each of the retrieved records was screened by two authors of this study (i.e. XZY, JTL) independently. If disagreement occurred between them, consensus on whether a study should be included was reached by discussion. In total, 20 papers (studies) written in English met our criteria. From each paper, the following information was extracted by the two above-mentioned authors of this study through full text screening independently: study site, sample type, sample size, methods used to characterize ARGs or ARs, and the main results or findings. When certain samples from non-mining-impacted environments (generally selected as controls) were also investigated by the targeted paper, these samples were excluded from our review. Again, in case of disagreement, consensus was reached by discussion between the two authors. We grouped the 20 targeted papers in Table S1 by the types of methods used to characterize ARGs or ARs, and summarized the broad findings of papers within each group (Fig. S1).

### A global-scale data collection

We performed a global-scale data collection on 15 July 2019. Specifically, the words of “acid mine drainage metagenome” or “mine water metagenome” or “mine tailings metagenome” were searched in the SRA database for raw reads and in the GenBank database for assemblies from NCBI (http://www.ncbi.nlm.nih.gov/). The SRA database coverage was 2007 to present, and the GenBank database coverage was 1982 to present. The retrieved 795 metagenomes were screened by two authors of this study (i.e. XZY, J-LL) independently as described above. Records with “whole-genome sequencing” as sequencing strategy were downloaded, and those with “Amplicon” or “RNA-seq” were filtered. As such, we obtained 50 metagenomes generated from HiSeq/MiSeq platforms (Illumina), 14 from 454 pyrosequencing, and two from ABI PRISM 3730 sequencer (Table S2). The metagenomic data from Illumina platforms were downloaded as raw reads, while those from 454 and ABI were downloaded as assemblies. The geographic information (location, latitude, and longitude) on each study site (generally containing several metagenomes) and sample description (sample size, sample type, and mine type) were retrieved from the sample information provided by the NCBI BioSample database. In total, 16 mine sites distributed globally (Fig. S2A) were represented by the 66 public metagenomes derived from our data collection. Although the ores in these mine sites are non-ferrous metal minerals, the major metals being mined differ considerably from site to site (Table S2). The latitude and longitude of these mine sites varied greatly from 6° 26′ 24″ S to 65°3′ 36″ N and from 122° 31′ 48″ W to 113°42′ 36″ E, respectively (Table S2). We also tried to retrieve the information on climate conditions of these mine sites and physicochemical properties of the samples, but such information was generally not available in the NCBI database.

### Two national sampling efforts

One nation-wide sampling effort was made during July and August 2018, in which a total of 39 mine sites distributed across China (Fig. S2B) were sampled. These mine sites covered a wide range of latitude and longitude (22° 8′ 19″ N–48°15′ 54″ N, 86° 19′ 47″ E–29° 17′ 29″ E; Table S3). The climatic conditions of them also varied considerably, with mean annual precipitation (MAP) of 25–1917 mm and mean annual temperature (MAT) of −0.09 to 22.8 °C (www.worldclim.org). At each mine site, three mine tailings samples were taken from a drained tailings pond (i.e. an abandoned tailings disposal site) at a depth of 0–20 cm using a stainless steel trowel. Specifically, three plots (1 × 1 m, separated from each other by at least 10 m) were set in each pond and one tailings sample was collected from each plot.

The other national sampling effort was made during July and August 2017, wherein 20 mine sites located across South China (Fig. S2C) were sampled. The latitude and longitude of these mine sites ranged from 22° 57′ 52″ N to 31° 40′ 39″ N and from 105° 43′ 43″ E to 118° 37′ 40″ E, respectively (Table S4). The MAP and MAT of them varied from 1110 to 1849 mm and from 10.1 to 20.0 °C, respectively. At each mine site, three to ten AMD sediment samples were collected from an AMD pond using a sediment collector at a depth of 0–10 cm. The sample size for a given pond was roughly proportional to its area and the sampling points for each pond were separated from each other by at least 10 m.

### Physicochemical analysis

All collected samples were transported back to the laboratory in an ice box within 24 h, homogenized for three minutes in a blender, and then divided into two parts. One part was air-dried for physicochemical analysis, and the other part was placed in a refrigerator at −20 °C for DNA extraction. Air-dried tailings and AMD sediment samples were analyzed by standard methods described previously for pH, electrical conductivity (EC), ferrous and ferric iron (Fe^2+^ and Fe^3+^), total carbon (TC), total nitrogen (TN), total phosphorus (TP) and sulfate (SO_4_^2−^) [23]. Total concentrations of metals (cadmium, copper, iron, lead, manganese, and zinc) in the samples were determined by an atomic absorption spectroscopy (AAS: AA-7000, Shimadzu, Japan) after being digested by a mixture of concentered HNO_3_/HCl (1:3). Bioavailable fraction of metals was analyzed by an AAS as well after extraction with diethylenetriaminepentaacetic acid. Total mercury was quantified by a cold vapor atomic fluorescence spectrometry (CVAFS: Tekran 2500, Tekran Inc., Canada) after digestion with a mixture of concentered HNO_3_/HCl (3:1). Methylmercury was measured by a gas chromatography (GC)-CVAFS (Tekran 2700, Tekran Inc., Canada) after preparation using CuSO_4_-methanol/solvent extraction [24].

### DNA extraction and shotgun metagenomic sequencing

For each tailings sample, 10–30 g of tailings were extracted for total genomic DNA using FastDNA Spin kit (MP Biomedicals, Santa Ana, CA, USA). For each sediment sample, 1–5 g of sediments were used for total genomic DNA extraction with PowerSoil DNA isolation kit (Mobio Laboratories Inc., Carlsbad, CA, USA) according to the manufacturer’s protocol. The extracted DNA yield and purity were evaluated by a NanoDrop 2000 spectrophotometer (Thermo Scientific, Waltham, MA, USA). The purified DNA from each sample was subsequently used to construct a sequencing library (~300 bp average insert size) with NEBNext Ultra II DNA PCR-free Library Prep Kit (New England Biolabs, Ipswich, MA, USA), and was shotgun-sequenced with MiSeq Reagent Kit v3 on the MiSeq platform with PE150 mode (Illumina, San Diego, CA, USA).

### Metagenomic assembly, binning and open reading frame (ORF) prediction

The metagenomic sequencing data were processed by our in-house pipelines to generate high-quality reads, including removal of duplication sequences, low-quality sequences (Q30), and sequences containing five excess Ns [25]. For the global public AMD-related metagenome dataset (hereafter referred to as “the Global-A dataset”) and the South China AMD sediment metagenome dataset (“the SChina-S dataset”), high-quality reads from each sample were individually assembled into contigs using SPAdes (version 3.9.0) with the parameters “-k 21, 33, 55, 77, 99, 127 –meta” [26]. For the China mine tailings metagenome dataset (“the China-T dataset”), high-quality reads of samples from the same mine site were co-assembled into contigs using MEGAHIT (version 1.2.9) with the parameters “k-min 35, k-max 95, k-step 20” [27]. For the SChina-S dataset, binning was performed with scaffolds longer than 2000 base pair (bp) using DAS Tool v.1.00 [28] with four binning methods: ABAWACA v.1.07 (https://github.com/CK7/abawaca), CONCOCT v.0.4.0 [29], MaxBin v.2.2.2 [30] and MetaBAT v.2.12.1 -[28, 31]. For the China-T dataset, scaffolds with length ≥ 2000 bp were retained and binning was carried out using MetaWRAP v.1.2.1 with three binning methods (CONCOCT v.0.4.0, MaxBin v.2.2.2, and MetaBAT v.2.12.1) [32]. The resulting MAGs were improved by RefineM v.0.0.25 [33] and further examined manually. The completeness and contamination of the refined MAGs were assessed using CheckM v1.0.12 [34]. Binning was not performed for the Global-A dataset, given that it contained a limited number of contigs/scaffolds with length ≥2000 bp.

Assemblies (i.e. scaffolds or contigs) downloaded from public sources and self-assembled scaffolds were processed together for ORF prediction. All the assemblies longer than 500 bp were used to predict ORFs with MetaProdigal (v2.6.3, set as -p meta) [35]. The predicted ORFs longer than 100 bp were clustered to generate a non-redundant (NR) gene catalog separately for each dataset. Clustering was conducted using CD-HIT (v4.6.8) under a criterion of 95% identity over 90% of the shorter ORF length (set as -c 0.95, -aS 0.9, -g 1, -d 0) [36], producing a total of 7.90, 59.6 and 37.0 million NR genes from the Global-A dataset, the China-T dataset and the SChina-S dataset respectively.

### Annotation and quantification of ARG, MRG, MGE, and plasmid

ARG annotation was performed on gene sequences using the recently published DeepARG software (v1.0.1, set as –align –type nucl –genes) [37]. DeepARG uses a deep learning algorithm for ARG annotation, which improves the annotation accuracy (especially for genes with low sequence similarity to the reference ARGs). Genes were aligned against the MEGARes 2.0 reference dataset to identify MRGs [38]. MEGARes 2.0 integrated BacMet database [39] to a well-organized hierarchical classification ontology. Using the UBLAST algorithm, genes were screened with minimum query coverage of 40%, identity of 70% and *e* value below 1 × 10^−5^ and top hits were classified according to the MRG hierarchy [40].

Reference sequences from ISfinder (accessed on 18 September 2019) [41], INTEGRALL (20 September 2019) [42] and the Transposon Registry (11 October 2019) [43] were integrated and de-replicated using CD-HIT at a criterion of 95% identity over 90% of the shorter gene length to construct a database of mobile genetic elements (MGEs, Table S5), which included 10,829 transposases, 2615 integrases containing 88 class 1 integron integrase (*intI1*) sequences, 848 resolvases, and 526 recombinases. Genes were aligned against the self-constructed MGEs database using blastx implemented in DIAMOND v. 2.0.9 [44] at a criterion of minimum query coverage of 40%, minimal identity of 25%, and e-value below 1 × 10^−5^, and top hits were annotated as corresponding MGEs [40, 45]. The PlasFlow software [46] with default setting was used to predict plasmid sequences for all ARG-carrying contigs.

Gene catalogs from the three datasets were combined for the purpose of cross-mapping in quantification. In order to reduce computational time, genes annotated as ARG, MRG and MGE were extracted from the combined gene catalog and de-replicated (using CD-HIT set as -c 0.95, -aS 0.9, -g 1, -d 0) to create an NR gene subset with totally 1.70 million genes. Clean reads of each metagenome were mapped back to the NR gene subset using Bbmap (v38.44, set as *k* = 14, minid = 0.95) to calculate the coverage [47]. The abundance of a given gene type/subtype was calculated using the following equation [48]:

$${{{{{{{\mathrm{Abundance}}}}}}}}\,\left( {{{{{{{{\mathrm{coverage}}}}}}}},\,{{{{{{{\mathrm{t}}}}}}}}/{{{{{{{\mathrm{Gb = }}}}}}}}\mathop {\sum}\limits_1^n {\frac{{N_{{{{{{\rm{mapped}}}}}}\,{{{{{\rm{reads}}}}}}} \times L_{{{{{\rm{reads}}}}}}/L_{{{{{{\rm{NR}}}}}}\,{{{{{\rm{gene}}}}}}}}}{S}} } \right)$$

where *n* is the number of NR genes annotated to that gene type/subtype, *N*_mapped reads_ is the number of reads mapped to the NR gene, *L*_reads_ is the sequence length of the reads, *L*_NR gene_ is the length of the NR gene, and S is the size of the metagenomic data (Gb).

### Quantification of crAssphage and phage ɸB124-14

The genomes of crAssphage (accession NC_024711.1) and phage ɸB124-14 (HE608841.1) were downloaded from NCBI. Clean reads of each metagenome were mapped against the two genomes using BBmap (set as minid = 0.97), respectively. The abundances of the two phages in each metagenome were calculated as described above.

### ARG-MRG and ARG-MGE co-occurrence analysis

To explore the co-occurrence patterns of ARGs and MRGs, we first determined the proportion of contigs carrying both ARG and MRG, which was expressed as the percentage of contigs carrying at least one ARG plus one MRG in the total ARG-carrying contigs. Moreover, the following two parameters introduced by Li et al. [49] were calculated on contig and MAG basis, respectively: (1) the average minimum distance (MetA_min_ (kb)), which was obtained through dividing the sum of distances of each ARG to its closest MRG by the number of corresponding ARG-MRG pairs; and (2) the nearest MRG composition (%), which was measured based on the composition of closest MRG (with distance <100 kb) types for each ARG in corresponding ARG-MRG pairs. Contig-based and MAG-based co-occurrence analyses were performed using ARG-MRG-carrying contigs longer than 1000 bp and high-quality (completeness ≥95% and contamination ≤5%) MAGs, respectively. The co-occurrence patterns of ARGs and MGEs were analyzed in a similar way to those of ARGs and MRGs.

### Phylogenetic tree construction, taxonomic assignment, and virulence factor analysis of MAGs

Selected ARG-carrying MAGs from the China-T dataset and the SChina-S dataset were used to construct phylogenetic trees by PhyloPhlAn [50], respectively. The Newick files with the best tree topology were uploaded to the Interactive Tree of Life online interface [51] for visualization and formatting. We preferred to use a simplified cladogram for better illustration, given that our aim was to show the taxonomic assignments of selected ARG-carrying MAGs and the ARG dispersal characteristics rather than the phylogenetic distances between these MAGs. Taxonomic assignment of the ARG-carrying MAGs was inferred from the phylogenetic trees constructed with the reference genomes using GTDB-Tk [52].

A comprehensive list of human pathogens which contained 1005 species names (Table S6) were compiled from the literature [53–61]. MAGs annotated to species level were used for the identification of their potential pathogenicity by matching their species names to the self-compiled human pathogen list (Table S6). MAGs matched to the pathogen list were referred to as potential pathogens (Table S7). The identified potential pathogens and 30 non-pathogens MAGs (Table S7) selected from the MAGs retrieved in this study by use of a random number table were further predicted for virulence factor (VF) genes using the “Vir” and “Tox” workflow of the PathoFact software [62].

### Quantifying the relative abundances of ARG-carrying MAGs

The relative abundances of selected ARG-carrying MAGs were calculated as previously described [25]. Briefly, the high-quality reads from each metagenome were mapped to all dereplicated MAGs using BBMap with the parameters k = 14, minid = 0.97 and build = 1. The coverage of each MAG was calculated as the average scaffold coverage, and each scaffold was weighed by its length in base pairs. Subsequently, the coverage of a given ARG-carrying MAG in each metagenome divided by the total coverage of all MAGs with completeness >50% and contamination <5% (irrespective of whether they carried ARGs or not) in the corresponding metagenome was considered as its relative abundance in that metagenome.

### Comparison of mine wastes, untreated sewage, and freshwater sediments

In order to make a direct comparison of mine sites and antibiotic-polluted environments in ARG profile, we collected public untreated urban sewage and freshwater sediment metagenomes. Untreated urban sewage was selected because it is a well-known ARG hotspot polluted by antibiotics [63] and can be considered as a “positive” control. A recent study by Hendriksen et al. [63] contained a total of 79 metagenomes of untreated urban sewage collected from 60 countries with the same method, providing us an excellent dataset for comparison. We thus selected 30 our metagenomes (Table S8) and 30 their metagenomes (Table S9) by use of a random number table for comparison. Freshwater sediment was chosen as a “negative” control. To that end, we performed another public data collection by searching “river sediment metagenomes” or “lake sediment metagenomes” against the SRA database on 27 October 2021. The search was then refined by choosing Source of “DNA”, Library layout of “paired”, Platform of “Illumina”, Strategy of “Genome” and File Type of “fastq”. Subsequently, metagenomes of “sediment enrichment” and “estuarine sediment” were further excluded. Finally, a total of 1305 records were obtained, from which 30 metagenomes were selected in a random manner as described above to download (Table S10). The relevant sample information available in NCBI showed that the corresponding sediments spanned a vast geographic area and that the majority of them were polluted by various anthropogenic activities (Table S10).

Raw reads of the 60 selected public metagenomes were downloaded and low-quality reads were filtered out as described above. The clean reads of these public 60 metagenomes, together with those of the 30 mine waste metagenomes selected for comparison, were first used for assessment of their microbial taxonomic diversity using Nonpareil software [64]. Give that the overall taxonomic coverage was higher in mine wastes than in the other two sample types at current sequencing depths (Fig. S3), clean reads of the 30 mine waste metagenomes were sub-sampled (Table S8) in order to obtain a data size equivalent to that of the other two sample types (Tables S9 and S10). Clean reads from each sample were individually assembled into contigs using SPAdes (version 3.9.0) and contigs were processed as described above. In order to achieve a fair comparison, we created an NR gene catalog containing ARGs only from the 60 selected public metagenomes and the 30 subsampled mine waste metagenomes for cross mapping to mitigate bias caused by data size.

All read-based taxonomic annotation was performed using Kraken2 [65]. Taxonomic diversity was calculated using R software 3.6.3 and the Vegan package [66]. To calculate taxonomic distribution of ARGs (or MRGs), reads mapped to ARGs (or MRGs) in the NR gene catalog was first extracted using BBmap [47] and further went through taxonomic annotation. The percentage of species carrying ARGs (or MRGs) in a given community (metagenome) was calculated by dividing the total number of species inferred from the ARG-like (or MRG-like) reads by the total number of species inferred from all reads of that metagenome.

### Statistical analysis

R software 3.6.3 (R Foundation for Statistical Computing) was used for the statistics analysis and plotting. Linear regressions between genes were based on log-transformed gene abundance using function *log1p*, while Pearson and Spearman correlation analyses were performed with function *cor.test*. Smoothing curves using a linear model were drawn with function *geom_smooth* in ggplot2. Multiple-group comparisons were performed using Kruskal-Wallis test and two-group comparisons were analyzed with two-sided Wilcoxon signed-rank test.

Variation partitioning analysis (VPA) based on partial redundancy analysis (RDA) was conducted using the vegan package [66] to evaluate the effects of environmental factors, including metal-related parameters, other physico-chemical parameters, and climatic as well as geographic factors (details presented in Tables S3 and S4), on the total abundance or composition of ARGs observed in the two national sampling efforts in China. Metal-related parameters included total and bioavailable fraction of cadmium, copper, iron, lead, manganese and zinc. Concentrations of total mercury and methylmercury were additionally tested in the SChina-S dataset. Besides, several metal contamination indexes were also considered as metal-related parameters. Other physicochemical parameters included pH, EC, TC, TN and TP. Climatic and geographic factors were considered as geographic location-related parameters.

Principal coordinate analysis (PCoA) was performed to evaluate the differences in ARG composition among samples based on the Bray–Curtis distance of ARG abundance. Anosim test and Adonis test were conducted to determine significance differences in ARG composition caused by geographic location. The distance decay of ARG composition similarity (defined as 1 - Bray–Curtis distance by ARG subtypes) was also analyzed by considering individual mine sites as sampling units. Geographic distance between sites was calculated with the R package geosphere v1.5.10 [67].

Bar, box, dot, heatmap and histogram graphs were plotted with ggplot2 package v.3.3.2 [68]. R package eulerr v.6.1.0 [69] was applied to plot Venn diagram. Maps were created with the aid of R packages including maps v.3.3.0, rgdal v.1.5.18 [70], mapproj v.1.2.7 [71] and maptools v.0.9.9 [72].

## Results

### The current state of knowledge about ARGs in mining-impacted environments

According to the types of methods used to explore ARGs or ARs, the 20 currently available studies explicitly addressing ARGs or ARs in mining-impacted environments can be divided into the following three groups: (1) culture-based, (2) qPCR-based, and (3) metagenomics-based (Table S1). The first group included 13 studies and investigated the ARs of a total of approximately 930 bacterial strains. Among these strains, 35.1% remained to be classified taxonomically and all the rest were affiliated to *Actinobacteria*, *Firmicutes* or *Proteobacteria* (Table S1 and Fig. S1A). Note that the three phyla have been reported frequently to be hosts of ARGs in various environments polluted by antibiotics [73]. The second group contained six studies and examined a limited number of ARGs (ranging from 14 to 65) in six mine sites. The six most abundant ARGs of these mine sites belonged to three different ARG types (i.e. macrolide, sulfonamide, and tetracycline; Table S1 and Fig. S1B). The third group with only one study analyzed ARGs in two mine sites and identified bacitracin resistance genes as the most dominant ARG type (Table S1 and Fig. S1C). Taken together, these findings of our scoping review indicate that the current knowledge on ARGs in mining-impacted environments is far from sufficient to allow a comprehensive understanding of the ARGs in such environments. This thus highlights the need for more research efforts to test our abovementioned hypotheses, which are critical to understand main features of the ARGs in mining-impacted environments but have not yet been addressed in the literature.

### Abundance, diversity, and composition of ARGs in mining-impacted environments

**Fig. 1:**
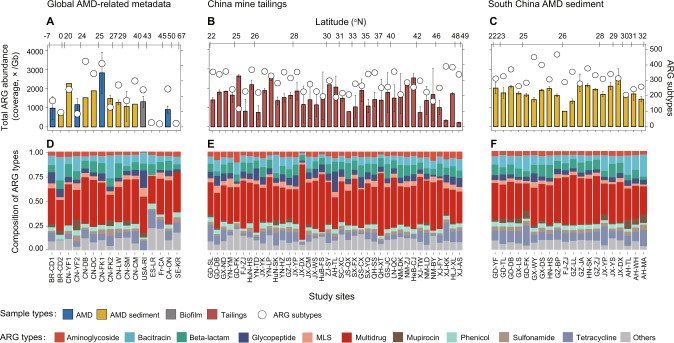
Abundance, diversity, and composition of ARGs in the mine sites distributed globally. Studied mine sites in each dataset are arranged on *X*-axis by latitude from south to north. Detailed information on the mine sites and samples are presented in Tables S2–S4. **A**–**C** Bars represent the total ARG abundances and circles represent the number of ARG subtypes, respectively. The total ARG abundances are expressed as coverage normalized to data size (×/Gb). Error bars represent standard deviation (s.d.) of samples in each mine site. **D**–**F** Compositions of ARG types in the mine sites. ARG types beyond the top 10 most abundant types are grouped into “Others”, with an average relative abundance < 2%. AMD and MLS are short for acid mine drainage and macrolide-lincosamide-streptogramin, respectively.

The Global-A dataset contained 66 public AMD-related (i.e. AMD, AMD sediment, and AMD biofilm) metagenomes from 16 mine sites in a total of seven countries, including Brazil, Canada, China, France, Spain, Sweden, and USA (Figs. 1A, S2A, and Table S2). Due to the difficulty in extraction of DNA from metal-rich environmental samples, the DNA extracted from two mine tailings and four AMD sediment samples collected in the two national sampling efforts did not meet the criterion for metagenomic sequencing, but these samples accounted for only 2.8% of the total samples. As such, the China-T dataset included 115 tailings metagenomes from 39 mine sites in 21 provinces across China (Figs. 1B, S2B, and Table S3) and the SChina-S dataset contained 91 AMD sediment metagenomes from 20 mine sites in seven provinces across South China (Figs. 1C, S2C, and Table S4). We did not merge the three datasets, given that they had some differences in sampling design and experimental methods.

The average data size of the Global-A dataset, the China-T dataset and the SChina-S dataset was 13.6 ± 12.3, 69.6 ± 14.0, and 75.2 ± 11.1 Gb clean reads per metagenome, respectively. Examination of a subset of 30 metagenomes from our national sampling efforts using Nonpareil indicated that the sequencing depth of our samples are largely sufficient to cover most taxonomic diversity (>80% coverage in all 30 samples and > 95% in 22 samples) in the mine wastes (Fig. S3A). A total of 25,575, 192,929, and 338,451 ORFs were annotated as ARG-like sequences in the three datasets, respectively. These ORFs were carried by 21,948, 175,175, and 302,841 ARG-carrying contigs (ACCs), correspondingly. Thirty-eight regulatory ARGs were identified and excluded from the following analyses in this study (unless explicitly stated).

The total abundances of ARGs (coverage, ×/Gb) in the three datasets were in the range of 814–2836 (with an average of 1444), 219–2632 (1516) and 806–2607 (1795), respectively (Fig. 1A-C). More specifically, the total abundances of ARGs in 76.9, 82.1, and 95.0% of mine sites in the three datasets were greater than 1000, respectively.

Twenty-eight ARG types were detected in the mine sites, consisting of 668, 723, and 660 ARG subtypes in the three datasets, respectively (Fig. 1A–C). Except for three European mine sites with no reads data where the numbers of detected ARG subtypes were < 28, the number of ARG subtypes in individual mine sites ranged from 85 to 468 (Fig. 1A-1C). Moreover, most of the studied mine sites (61.5% in the Global-A dataset, 89.7% in the China-T dataset and 100% in the SChina-S dataset) harbored more than 200 ARG subtypes.

The composition of ARG types was fairly consistent across all studied mine sites (Fig. 1D–F). Multidrug, bacitracin, beta-lactam, tetracycline, and glycopeptide were the top five most dominant ARG types. Among them, multidrug was the most dominant ARG type across all samples, accounting for an average of 39.7, 41.6, and 38.7% of the total ARG abundance in the three datasets respectively. In several extreme cases (i.e. nine mine sites in the China-T dataset), the relative abundances of multidrug resistance genes were beyond 50%, with a maximum of 76.3%. Similarly, multidrug was shown to be the most dominant plasmid-carrying ARG type (Fig. S4). Antibiotic efflux and antibiotic target alteration were the most dominant resistance mechanisms across all the mine sites (Fig. S5).

When abundant ARG subtypes were defined as those with an average relative abundance > 1% in all samples in a given dataset, 24, 19, and 20 abundant ARG subtypes were observed in the three datasets, respectively (Fig. S6A–C). After de-duplication, a total of 33 abundant ARG subtypes were found. Among them, 15 and two belonged to multidrug and bacitracin resistance genes, respectively. When ubiquitous ARG subtypes were defined as those occurred in all samples in a given dataset, 15, 33, and 81 ubiquitous ARG subtypes (Fig. S6D–F; detailed information listed in Table S11) were found in the three datasets respectively, which had high overlaps with the corresponding abundant ARG subtypes. Among the ubiquitous ARG subtypes in the three datasets, 5 (33.3%), 14 (42.4%), and 31 (38.3%) were multidrug resistance genes (Fig. S6D–F).

**Fig. 2:**
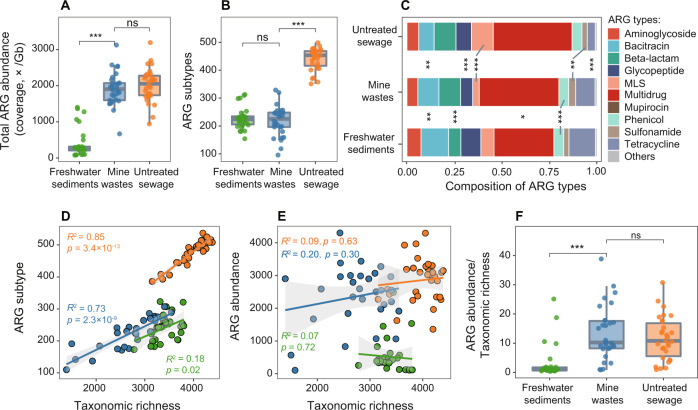
Comparison of ARG profiles and taxonomic diversity in mine wastes, untreated urban sewage, and freshwater sediments. **A** The total ARG abundances in the three types of environmental samples. **B** The numbers of ARG subtypes in the three types of environmental samples. The boxes represent 25th percentile, median and 75th percentile of the data, and the whiskers show the minimum or maximum value of the data. **C** Compositions of ARG types in the three types of environmental samples. Comparison between two types of samples was analyzed with Wilcoxon signed-rank test. ns: non-significant; *p < 0.05; **p < 0.01; ***p < 0.001. Non-significant differences between sample types in some ARG types were not labeled. Detailed information on the samples is presented in Tables S8–S10. **D** Pearson correlations between ARG diversity (ARG subtypes) and microbial taxonomic richness. **E** Pearson correlations between ARG abundance and microbial taxonomic richness. Taxonomic richness was inferred from reads and calculated on the species level. **F** ARG abundance normalized by taxonomic richness in the three types of environmental samples. Colors: green for freshwater sediments, blue for mine wastes, and orange for untreated sewage.

The analysis based on a subset of our metagenomes and public metagenomes showed that the average total abundance of ARGs in mine wastes rivaled that of untreated urban sewage but was 3.7 times higher than that of freshwater sediments (*p* < 0.001, Fig. 2A). The number of ARG subtypes was higher in untreated urban sewage than those in mine wastes and freshwater sediments (*p* < 0.001, Fig. 2B). The percentage of ARGs classified as multidrug resistance genes in mine wastes was comparable to that of untreated urban sewage, being significantly greater than that of freshwater sediments (*p* < 0.05, Fig. 2C). Despite this, the relative abundances of other five abundant ARG types (e.g. bacitracin, MLS) differed significantly (*p* < 0.01, Fig. 2C) between mine wastes and untreated urban sewage, thus supporting our second hypothesis.

ARG diversity (subtype number) was positively correlated with microbial taxonomic diversity in all three sample types (*p* < 0.05; Fig. 2D), being comparable to those findings reported previously for various sample types [74–76]. No significant correlations were seen between microbial taxonomic diversity (i.e., richness) and the total abundance of ARGs in all three sample types (Fig. 2E). This result was inconsistent with the negative microbial diversity–ARG abundance relationship observed in a microcosm experiment [77], suggesting that environmental factors may have a more important role than microbial diversity in determining ARG abundance in real environmental samples. On average, the abundance of ARGs per microbial species was much higher in mine wastes and untreated sewage than in freshwater sediments (*p* < 0.001, Fig. 2F), which was likely attributed to the higher selective pressures associated with the former two sample types [6, 76]. Similar results were recorded for MRGs (Fig. S7).

On average, 55.0% of the microbial species in mine wastes carried ARGs, and the corresponding figures were 59.2% in untreated sewage and 49.1% in freshwater sediments (Fig. S8A). Multidrug resistance genes were more widely spread in the microbial community than other ARG types for all three sample types, especially untreated sewage and mine wastes (46.6 and 43.1% respectively; Fig. S8B–L). Similar taxonomic distributions were observed for MRGs, wherein Cu and multimetal resistance genes were among the most widely spread MRG types (Fig. S9).

### Factors determining the total abundance of ARGs in mining-impacted environments

**Fig. 3:**
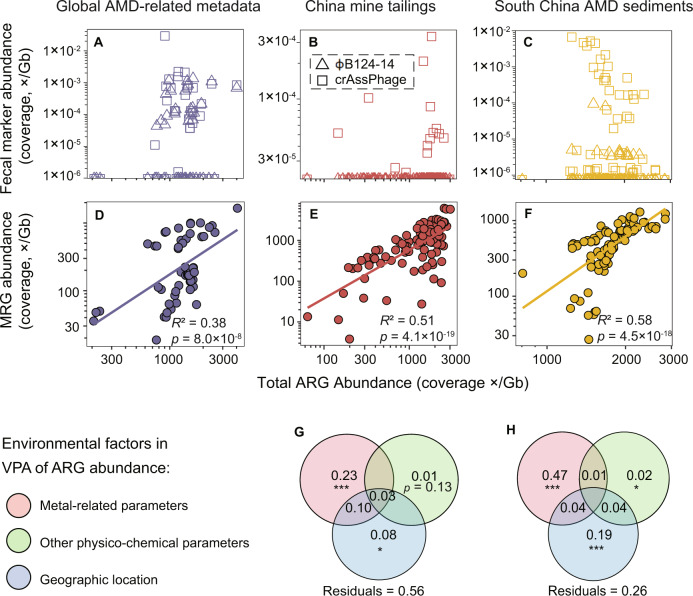
Potential factors shaping ARGs in the studied mine sites. **A**–**C**. Scatterplots of the fecal marker gene (φB124-14 phage and crAssPhage) abundances versus the total ARG abundances. Regression lines, *r* and *p* values are not shown because correlations were not significant (*p* > 0.05). Correlations were calculated based on log-transformed abundances. **D**–**F**. Pearson correlations between the total metal resistant gene (MRG) abundances and the total ARG abundances. All gene abundances are expressed as coverage normalized to data size (×/Gb). **G** and **H**. Variations of ARG abundances explained by environmental factors in the China-T (**G**) and SChina-S dataset (**H**). VPA is short for variation partitioning analysis. Statistical significance for each part of variation explained was checked using ANOVA test with 999 permutations. *: *p* < 0.05; ***: *p* < 0.001. Detailed information on environmental variables is presented in Tables S3 and S4. VPA was not performed for the Global-A dataset due to the lack of relevant data.

Due to the hazards of mine wastes to human and environmental health, their disposal sites generally are located in mountain valleys that are far away from human settlements [14]. In this context, there is a low probability that the enrichment of ARGs in such sites is associated with fecal or other contamination sources (e.g. agricultural runoff). To be on the safe side, however, two fecal markers (phage ɸB124-14 and crAssPhage) were quantified. Both markers were detected at low frequency and low abundance (Fig. 3A–C). For instance, none of the mine sites in the China-T dataset contained phage ɸB124-14 and only 13.9% of the studied mine sites contained crAssPhage with a total abundance below 3.4 × 10^−4^ (coverage, ×/Gb). Nonetheless, no significant correlations were found between the total abundance of either ɸB124-14 or crAssPhage and that of ARGs in all the three datasets. The total ARG abundance was either not correlated with or weakly correlated with that of *intI1* genes (Fig. S10).

MRGs were abundant in most of the mine sites (Fig. S11). More specifically, 30.2, 71.8 and 54.2% of the mine sites in the three datasets contained MRGs with a total abundance beyond 500 (coverage, ×/Gb). Moreover, the total abundances of MRGs in mine sites in all three datasets were highly correlated to those of ARGs (*p* ≤ 8.0 × 10^−7^, Fig. 3D–F).

A total of 44 and 74% of the variations of total ARG abundance in the China-T dataset and the SChina-S dataset were explained by the three groups of environmental factors (Fig. 3G, H). Among them, the metal-related parameters were the most important, as they alone explained 23 and 47% of the total variations in the two datasets respectively (*p* < 0.001). Note that another 10 and 4% of the total variations in the two datasets were explained by the metal-related parameters due to their collinearity with geographic factors, respectively. Geographic factors alone explained only 8 and 19% of the total variations in the two datasets respectively (*p* < 0.05), and other physico-chemical parameters alone explained only 1 and 2% correspondingly. Pearson correlation analysis showed that zinc and available manganese concentrations exhibited the highest correlation with the total ARG abundance among the metal-related parameters in the two datasets, respectively (*p* < 0.05, Fig. S12).

### Co-occurrence patterns of ARGs and MRGs in mining-impacted environments

**Fig. 4:**
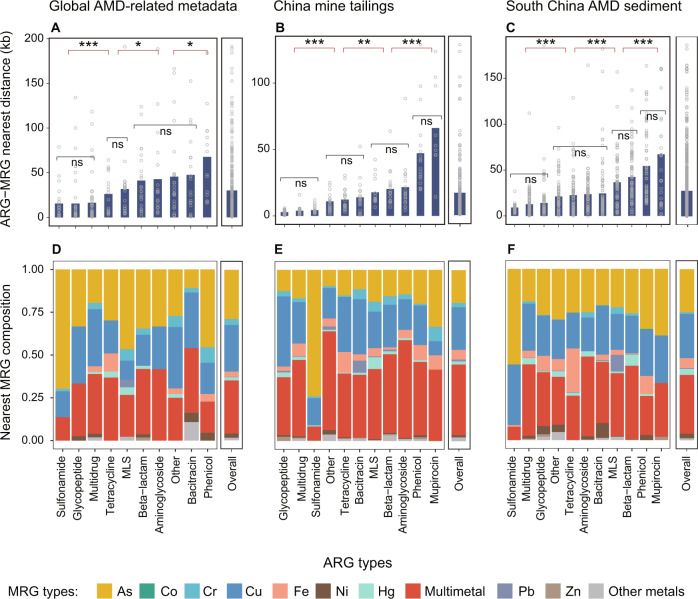
Co-occurrence patterns of ARGs and MRGs in the studied mine sites. **A**–**C** Contig-based average nearest distance between ARGs and MRGs. The medium value for each sample is indicated by a hollow dot. Comparison of two groups was analyzed with Wilcoxon signed-rank test and comparison of multiple groups was analyzed with Kruskal–Wallis test. ns: non-significant; *p < 0.05; **p < 0.01; ***p < 0.001. **D**–**F**. Compositions of nearest MRG types for each ARG type. The top ten most abundant MRG types are presented. The other MRG types were detected with an average relative abundance below 0.3% and thus are referred to as “Other metals”.

On average, the proportions of ACCs which also carried at least one MRG to all ACCs in the three datasets were 9.5, 9.1 and 9.0%, respectively (Fig. S13). Multidrug, glycopeptide and sulfonamide, with average MetAmin to MRGs of 12.7, 9.5 and 13.1 kb, respectively, were among the closest ARG types toward MRGs (*p* < 0.001, Fig. 4A–C; contig-based results). On the contrary, phenicol and mupirocin resistance genes were the farthest to its closest MRGs (Fig. 4A–C). Similar results were obtained from the MAG-based analysis (Fig. S14).

For all ARG types in the three datasets, their nearest MRG compositions were considerably consistent: on average 33.7% (with a range of 7.7–57.6%), 27.1% (3.9–74.0%) and 23.6% (8.3–41.0%) of their closest MRGs were multimetal, arsenic and copper resistance genes, respectively (Fig. 4D–F). Taking multidrug resistance genes as an example, 34.9% of their closest MRGs in the Global-A dataset were multimetal resistance genes, while those figures for the other two datasets were 44.6 and 41.6% respectively. This preferred connection between multidrug resistance genes and multimetal resistance genes was also seen in similar analyses based on MAGs (Fig. S14) and plasmids (Fig. S15).

### Mobility of ARGs in mining-impacted environments

**Fig. 5:**
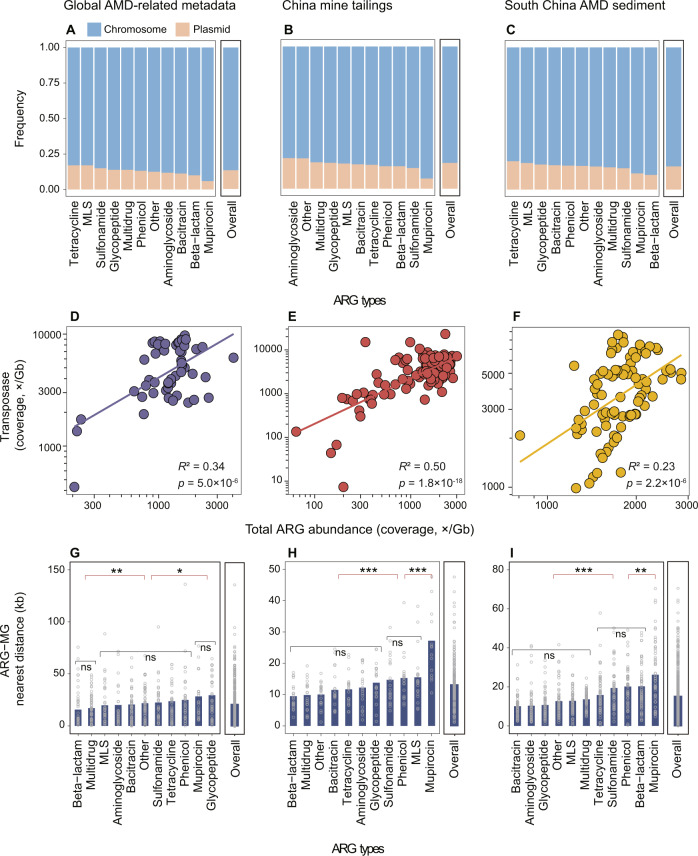
Mobility of ARGs in the studied mine sites. **A**–**C**. Proportions of ARG-carrying contigs encoded by plasmids and chromosomes. Contigs which could not be identified as either plasmid or chromosome were excluded from the calculation. **D**–**F**. Pearson correlations between log-transformed abundances of transposase genes and the total ARG abundances. **G**–**I**. Contig-based average nearest distance between ARGs and MRGs. Variations between two groups were analyzed with Wilcoxon signed-rank test and multiple-group comparisons were analyzed with Kruskal–Wallis test. ns: non-significant; *p < 0.05; **p < 0.01; ***p < 0.001.

An average of 13.2, 18.1, and 16.2% of ACCs in the three datasets were annotated as plasmids, respectively (Fig. 5A–C). When multidrug resistance genes were taken into account, 13.8 to 18.5% (on average 16.0%) of these dominant ARGs in the three datasets were located on plasmids respectively. Although the majority of ARGs were located on chromosomes, plasmids were still enriched ARG carriers in terms of ARG-carrying density. Specifically, an average of 16 ARGs were found within every 100 kb plasmid sequences, while that figure for chromosome sequences was five (Fig. S16). Note that on average 24.5, 15.1, and 24.0% of the total ARG-carrying plasmids in the three datasets were found to harbor at least one MGE (Fig. S17).

The total abundances of transposases (coverage, ×/Gb; with an average of 4311) were much higher than those of integrases, recombinases and resolvases (Fig. 5D–F, S18). More importantly, significant positive correlations between the total abundance of transposases and that of ARGs were found in all three datasets (*p* < 0.001, Fig. 5D–F). Similar correlations were also observed for the other three MGE types and ARGs (Fig. S18).

Co-occurrence patterns of ARGs and MGEs were examined in terms of their nearest distances on the same contigs (Fig. 5G–I). Multidrug resistance genes were among the ARG types that were located closest to MGEs, with average MetA_min_ at 17.2, 9.9, and 13.7 kb in the Global-A dataset, the China-T dataset, and the SChina-S dataset, respectively (Fig. 5G–I).

### Biogeography of ARGs in mining-impacted environments

**Fig. 6:**
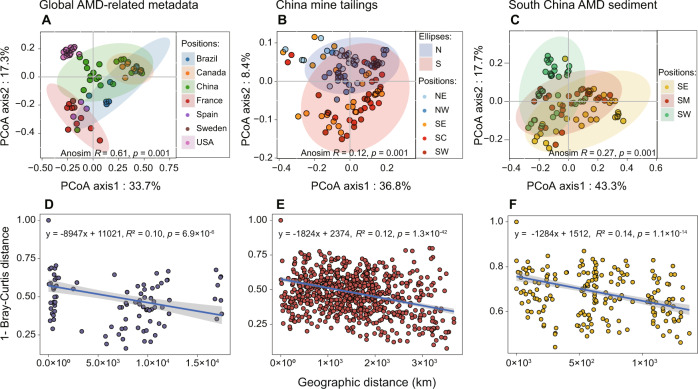
Geography of ARGs in the studied mine sites. **A**–**C** Principal coordinates analysis (PCoA) of ARG composition based on Bray-Curtis dissimilarity grouped by geographic position. N north, S south, NE northeast, NW northwest; SE southeast; SC south central; SW southwest. **D**–**F** Spearman’s rank correlations between the Bray–Curtis similarity of ARG composition in the studied mine sites and the geographical distance between mine sites.

Our PCoA showed that ARG composition in the studied mine sites tended to be clustered by geography (Fig. 6A–C): (1) mine sites in the Global-A dataset were grouped roughly according to country; (2) most mine sites in the China-T dataset could be divided into two groups (i.e. South (S) and North (N) China, respectively); and (3) those in the SChina-S dataset were generally clustered into three groups, including south-east (SE), south-central (SC) and south-west (SW) China. Although the clustering pattern observed at the global scale could be magnified by different experimental methods of different studies, our distance-decay analysis also revealed that geography had a significant influence on ARG composition similarities in all three datasets (*p* < 0.001, Fig. 6D–F). Similar as what were observed in the PCoA and distance decay analysis, VPA suggested that geographic location alone or in combination with the metal-related parameters significantly affected ARG compositions of the China-T dataset and the SChina-S dataset (*p* < 0.001, Fig. S19), while such an analysis was not applicable to the Global-A dataset due to the lack of relevant data. These results supported our fourth hypothesis.

### Hosts of ARGs in mining-impacted environments

**Fig. 7:**
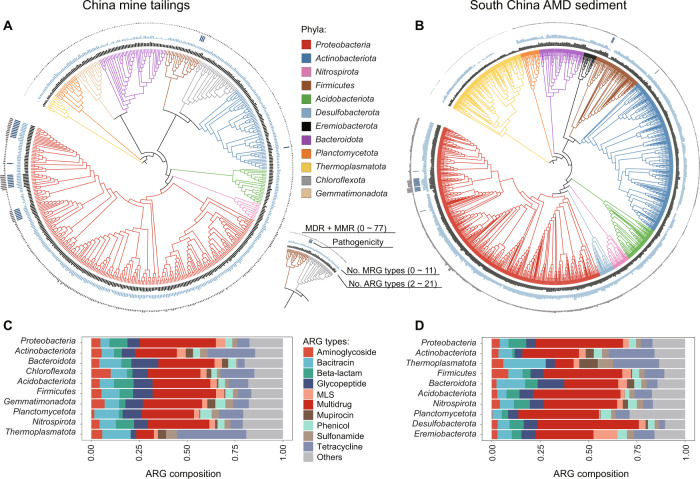
Hosts of ARGs in the studied mine sites. **A,** **B** Phylogenetic trees of the top 10 dominant ARG-host phyla in terms of the number of high-quality ARG-carrying metagenome-assembled genomes (MAGs: ≥95% completeness and ≤5% contamination) affiliated to each phylum. The maximum-likelihood phylogenetic trees were constructed using PhyloPhlAn and shown as cladograms. The first two layers indicate the number of ARG and MRG types possessed by each MAG, respectively. The third layer denotes the pathogenicity of each MAG (presence or absence). Pathogenicity identification was made to those MAGs annotated to species level. The outside layer shows the total number of multidrug resistance genes (MDR) and multimetal resistance genes (MMR) carried by each MAG. **C**, **D** Compositions of ARG types carried by the top 10 dominant ARG-host phyla.

A total of 1800 and 5104 good-quality ARG-carrying MAGs (completeness ≥75% and contamination ≤10%) were recovered from the China-T dataset and the SChina-S dataset, respectively (Tables S12 and S13). They were affiliated to 41 phyla and carried 577 ARG subtypes (accounting for 73.5% of the total ARG subtypes identified in this study). Over 73.7% of them had ≥10 ARGs and 35.2% of them harbored more than 10 ARG types (Table S14). However, in order to improve the credibility and display quality of our results, hereafter we focused on 1830 high-quality ARG-carrying MAGs (representing 31 phyla), of which 565 and 1265 were from the China-T dataset and the SChina-S dataset respectively (Tables S12 and S13). In both datasets, *Proteobacteria* contained the largest number of ARG-carrying MAGs (Fig. 7A, B). As such, their relative abundances in most studied mine sites were higher than those of the other dominant phyla carrying ARGs (Fig. S20). Apart from *Proteobacteria*, *Acidobacteriota, Actinobacteriota, Bacteroidota, Firmicutes, Nitrospirota, Planctomycetota* and *Thermoplasmatota* were among the top 10 dominant ARG-carrying phyla in both datasets (Fig. 7A, B).

Each of the ARG-carrying MAGs in *Proteobacteria*, on average, harbored 12.1 ARG types (including 24.2 subtypes) and 4.7 MRG types (12.6 subtypes), being higher than those of the other dominant phyla (Fig. 7A, B). Despite this, ARG compositions of the top 10 dominant ARG-host phyla were highly consistent (Fig. 7C, D), with multidrug being the most dominant ARG type for nine phyla. Indeed, multidrug resistance genes were found to have a broad taxonomic distribution in both datasets. For example, they were detected in 23 phyla, 155 families and 176 genera in the China-T dataset (Fig. S21). Compared to other dominant phyla, the archaeal phylum *Thermoplasmatota* possessed a distinct ARG composition, with tetracycline and bacitracin being the top two dominant ARG types (Fig. 7C, D). Similar analyses at both family and genus levels also revealed certain taxa with a distinct ARG composition. For instance, bacitracin was a predominant ARG type for the archaeal genus *Acidiplasma* (Fig. S22).

In the two national datasets, 75 and 121 ARG-carrying MAGs could be annotated at species level (Fig. S23). Among them, 31 and 23 were identified to be potential pathogens, including *Pseudomonas putida, Stenotrophomonas maltophilia, Klebsiella pneumonia*, and so on (Figs. 7A, B, S23). These potential pathogens contained a significantly higher number of multidrug resistance, multimetal resistance and VF genes than the non-pathogens (*p* < 0.001, Figs. 7A, B and S24, S25).

## Discussion

### ARGs are abundant and overrepresented by multidrug type

As traditional abiotic pollutants, metals in mining-impacted environments have caused worldwide concern during the past decades [14, 16, 17]. However, their effects on the emergence, proliferation, and transmission of the new biotic pollutants ARGs in mine sites remain poorly understood. This study provides robust evidence for the first time that the proliferation and spread of ARGs in mining-impacted environments is an alarming issue on a global scale. On the one hand, the total abundances of ARGs in the mine wastes were not only much higher than those in the freshwater sediments with different extents of pollution but also comparable to those in the untreated urban sewage (Fig. 2A), a well-recognized ARG hotspot [63].

On the other hand, multidrug was found to be a predominant ARG type across all studied mine sites, on average contributing to 40% of the total ARGs in the mining environments (Fig. 1D–F). Such a ratio was higher than that of the freshwater sediments examined in this study and rivaled that of the untreated urban sewage (Fig. 2C). These findings are different from those of previous relevant studies (Fig. S1 and Table S1) and deserve much more attention, especially given that the increasing occurrence of multidrug-resistant pathogenic bacteria around the world has been considered as a major challenge in disease control [2]. Although there is evidence that some multidrug ARGs do not necessarily endow microorganisms the ability to tolerate multiple antibiotics [78], the dominance, drivers, and fate of multidrug ARGs in widely distributed environments represent an underexplored, but important topic.

It should be noted that the ARG subtype number in the mine wastes was only half of that in the untreated urban sewage (Fig. 2B). On the one hand, this may be partly due to the lower microbial taxonomic diversity in the mine wastes, given the strong correlation between microbial taxonomic diversity and ARG subtype number observed (Fig. 2D). On the other hand, the lower ARG subtype number in the mine wastes may as well be due to the lack of targeted metagenomes for some important mining countries (such as Australia, Chile, and Mexico) at the time when we performed our global-scale data collection. In such a context, we expect that the growing availability of metagenomes from mining-impacted environments worldwide will allow the identification of more ARG subtypes in the near future.

### Metals cause on-site selection of ARGs

There is emerging evidence that fecal contamination can largely explain ARG abundances in many anthropogenically impacted environments (e.g. sewage-polluted environments), without clear signs of on-site selection of ARGs [79]. However, in this study, we demonstrate that metals cause on-site selection of ARGs in mining-impacted environments, supporting our first hypothesis. First, the strong correlation between the total abundance of ARGs and that of MRGs (Fig. 3D–F) indicated the importance of co-selection with MRGs in ARG proliferation in the mine sites. Second, VPA analysis (Fig. 3G, H) and positive correlations between metal levels and total ARG abundance (Fig. S12) revealed that metals played important roles in driving ARG spread in the mine sites. Third, the low total abundances of fecal marker genes, in combination with the lack of correlation between their abundance and that of ARGs (Fig. 3A–C), ruled out the possibility that fecal contamination was a major source of the ARGs [79].

Metals are thought to co-select for ARGs through three mechanisms: cross-resistance, co-regulation, and co-resistance [6]. Our results revealed that all three mechanisms existed in mining-impacted environments. First, a large portion of ORFs were annotated as ARGs and MRGs simultaneously (Table S15), implying the importance of cross-resistance. Specifically, these ‘bifunctional’ ORFs consisted of 24 different ARG subtypes (Table S16), accounting for approximately 34.0% of the total ARG abundance (including regulatory ARGs, Table S16). Second, 11 of the 24 “dual-resistance-genes” were identified as regulatory genes (Table S15), some of which were detected with relative abundance >1% (e.g. *ArlR*, *ompR* and *vanR*; Table S16). These regulator genes indicate that co-regulation may be also an important mechanism. Similar inference about the importance of co-regulation in ARG co-selection has been made in an arsenic-spiking study [80]. Third, strong correlations between the total abundance of MRGs and that of ARGs were still observed even after removing the dual-annotated ARGs (Fig. S26), hinting the importance of co-resistance.

### Multidrug ARGs prefer to co-occur with multimetal MRGs

Although co-selection for ARGs and MRGs by metals has been observed in various environments [9, 12], a comprehensive understanding of the phenomenon is still lacking. To our knowledge, there are only two studies explicitly exploring the genetic relationships between ARGs and MRGs by taking advantage of public fully-sequenced bacterial genomes [49, 81]. Compared to the two studies, our study uncovered three distinct features of genetic linkages between ARGs and MRGs in mining-impacted environments. First, the ARG-MRG nearest distances in the mine sites (on average at 26.3 kb, Fig. 4A–C) were much shorter than not only that of non-pathogen genomes from diverse habitats (380 kb) but also that of pathogenic ones (103 kb) [49], providing evidence for our third hypothesis. Second, multidrug was always among the closest ARG types towards MRGs (Fig. 4A–C). Third, the most dominant co-occurring ARG-MRG pairs in the mining-impacted environments were multidrug ARG and multimetal MRG pairs (Fig. 4D–F). In contrast, based on a similar analysis of 5436 complete genomes of bacteria from diverse habitats, Li et al. [49] showed that beta-lactam, kasugamycin, bacitracin, aminoglycoside, polymyxin, and tetracycline were the top six ARG types that were most likely to co-occur with MRGs. One possible cause for such a discrepancy is that serious metal pollution in the mine sites exerts a directional evolutionary force in the co-selection for ARGs and MRGs [16]. These distinct features raise a possibility that mining-impacted environments are a pool of potential multi- antibiotic and metal resistant prokaryotes, given that close physical linkage of genes on genome can confer great advantage in developing corresponding phenotypes [82]. This possibility seems to be supported by an observation that 11 out of 16 microbial strains isolated from metal-polluted soils exhibited dual resistance to multiple metals and antibiotics [83].

### Multidrug ARGs are highly mobile

The mobility of ARGs is an important aspect of assessment and management of their environmental risk [76]. In this study, we observed that a large proportion of the ARGs were located on chromosomes rather than on plasmids (Fig. 5A–C). Despite this, the average proportion of plasmid-associated ARGs in the mine sites (approximately 16.0%) was still much higher than that of three wastewater treatment plants in Taiwan (ca. 5.0%) [84]. Note also that the proportion of plasmid-associated ARGs showed small variation among ARG types (Fig. 5A–C). In contrast, a previous study reported that plasmid-borne ARGs accounted for 0.0% to more than 90% of the total ARGs in coastal beach and sewage waters from Montevideo depending on ARG types [85]. The causes and potential implications of such a discrepancy deserve further investigation, although comparison between studies should be interpreted with caution because different plasmid identification methods were applied.

Two pioneer studies have consistently revealed a strong positive correlation between the total abundance of ARGs and that of transposase genes, thus highlighting the important role of horizontal gene transfer in the dissemination of ARGs in the hotspots [86, 87]. In agreement with this, we found that the total abundance of ARGs in the studied mine sites was positively correlated with not only that of transposase genes (Fig. 5D–F) but also those of genes encoding other three types of MGEs (Fig. S18). Moreover, the average nearest ARG-MGE distance in the mine sites was only 17.4 kb (Fig. 5G–I), which falls well within the active ranges of typical MGE types such as transposon (2.5–60 kb) [88], integrative and conjugative elements (11.5–155 kb) [89] and integrative and mobilizable elements (<50 kb) [90]. These findings are consistent with our third hypothesis. Among all ARG types detected in this study, multidrug ARGs were often featured with their shortest distance with MGEs (Fig. 5G–I), indicating that this ARG type had the highest dissemination potential in mining-impacted environments.

### Biogeographic analysis reveals ubiquity of multidrug ARGs

Geographic clustering was observed in both the China-T dataset and the SChina-S dataset (Fig. 6B, C). However, two previous studies showed that ARG compositions of urban landfill leachates and untreated urban sewage across China did not exhibit obvious geographic clusters [91, 92]. Such a discrepancy may be attributed partly to a scenario that mine sites are basically natural ecosystems with a more open environment as compared to various urban ecosystems and thereby are more susceptible to geographic factors. Likewise, we found that ARG composition of the public AMD-related samples varied considerably among countries (Fig. 6A), whilst a recent study showed that untreated urban sewage samples from 60 countries around the world were separated into only two groups in terms of their ARG composition [63]. Although we cannot exclude the possibility that using the same methodology is a reason for the weaker geographic grouping trend in the global sewage study, we showed that geographic factors had a significant effect on composition of the ARGs in mine sites even when the effect deriving from the collinearity between them and metal-related environmental factors was excluded (Fig. S19).

Irrespective of whether geographic factors significantly affect ARG composition, for some other well-known ARG hotspots, a small number of ARG subtypes tend to occur abundantly in almost all focal sites and thus are often referred to as core ARGs [12, 63, 84, 87, 91]. These core ARGs were also seen in the mine sites worldwide (Fig. S6). However, there exists a difference between the mining-impacted environments and other ARG hotspots in core ARGs. Specifically, while most core ARGs of untreated urban sewage in China or around the world endow resistances to several specific types of antibiotics (especially aminoglycoside and tetracycline) [63, 84, 87, 91], their counterparts of the mine sites belong mainly to multidrug ARGs (Fig. S6). Moreover, some of these multidrug ARGs (e.g. *comD, emrB* and *ompR*) were carried by potential pathogens (Table S17), being consistent with the information available in a public comprehensive ARG database wherein many hosts of these multidrug ARGs are considered as potential pathogens [93].

### Novel and potential pathogenic ARG hosts deserve more attention

Although the identification of ARG hosts is a critical step in developing strategies for reducing the spread rate of ARGs and antibiotic resistant pathogens, limited information about ARG hosts (especially those cannot be readily cultured) in the environment is available [73]. Our study uncovered immense diversity of environmental microorganisms that carried ARGs. Among the top 10 dominant ARG-host phyla in the mine sites (Fig. 7A, B), *Proteobacteria*, *Firmicutes*, *Bacteroidota,* and *Actinobacteriota* were reported frequently to occur in many other ARG hotpots [73]. However, to our knowledge, no complete genomes or MAGs belonging to *Thermoplasmatota*, *Gemmatimonadota*, *Desulfobacterota,* and *Eremiobacterota* have been previously found to carry ARGs [73], providing important support for our fifth hypothesis. *Thermoplasmatota* is a phylum affiliated to the Archaea domain, wherein antibiotic resistance remains poorly understood. There are only a few previous studies that identified methanogenic archaea as ARG hosts by network analysis [94, 95]. Thus, archaeal ARGs are of considerable interest, especially given that some archaea colonizing human microbiota have been reported to be implicated in diseases [96].

In this study, 97% of high-quality MAGs were found to carry at least one ARG, which was much higher than the figure reported by a previous study showing that 48% of the 5436 complete bacterial genomes downloaded from the NCBI genome database with diverse sources of habitat (including human, soil, and water) were ARG carriers [49]. One possible reason for our finding lies in that horizontal gene transfer across phylogenetic boundaries is more common in mining-impacted environments than in many others [97]. However, this does not fully explain the distinct ARG composition of *Thermoplasmatota* (Fig. 7C, D). Further research is warranted to explore the evolutionary relationships between the archaeal and bacterial ARGs. Nonetheless, our study suggests that in the mine sites a majority of dominant ARG-hosts prefer to harbor multidrug ARGs (Fig. 7C, D) and that this type of ARGs has the broadest host range (Fig. S21), which have not yet been observed in other environments [73].

Additionally, the 54 MAGs identified as potential pathogens in this study harbored more VF, multidrug resistance and multimetal resistance genes than the non-pathogens (Figs. 7A, B, S24, S25). Given the extremely low abundances of the fecal marker genes in the mine sites (Fig. 3B, C), we speculate that these potential pathogenic MAGs are not likely of human origin. Therefore, they should be referred to as “environmental pathogens” – organisms that normally spend a substantial part of their lifecycle outside human hosts, but when introduced to susceptible humans may cause disease with measurable frequency [98]. Note that, a few potential pathogens identified here, such as *P. putida*, *S. maltophilia* and *Pseudomonas aeruginosa*, have been reported to be soilborne or waterborne [99–102]. Nonetheless, the actual virulence of these potential pathogens inhabiting mining-impacted environments deserves further investigation.

## Conclusions

This study provides the first metagenomic evidence for serious pollution with ARGs in globally distributed mining-impacted environments, highlighting the distinct ARG characteristics of such environments as compared to other known ARG hotspots. While metatranscriptome-, qPCR- and/or culture-based studies are needed to further confirm our findings, we advocate to take effective measures to reduce the spread of ARGs from mine sites worldwide to their neighboring ecosystems. We also propose long-term monitoring of changes in ARGs and their hosts (especially those potential pathogens carrying both multidrug ARGs and multimetal resistance genes) in mine sites.

## Supplementary information

Supplementary figures

Supplementary table

## Data Availability

All data are available in the main text or the supplementary materials. The gene catalogue of ARG, MRG and MGEs has been deposited in NCBI BioProject database under accession code PRJNA847005. R scripts used in data analysis in this study can be found in the following github repository: https://github.com/anotherXinzhu/MiningResistome.git.
