# Supplementary material for: Globally distributed mining-impacted environments are underexplored hotspots of multidrug resistance genes
Source: ISME J. 2022 Jun 10;16(9):2099–113. doi: 10.1038/s41396-022-01258-z (PMC9381775; doi:10.1038/s41396-022-01258-z)

*Supporting Information for*

**Globally distributed mining-impacted environments are underexplored hotspots of multidrug resistance genes**

Xinzhu Yi^1,†^, Jie-Liang Liang^1,†^, Jian-Qiang Su^2^, Pu Jia^1^, Jin Zheng^1^, Jing-Li Lu^1^, Zhang Wang^1^, Shi-wei Feng^1^, Zhen-hao Luo^2^, Hong-xia Ai^2^, Bin Liao^2^, Wen-sheng Shu^1,3^, Jin-tian Li^1,*^, & Yong-Guan Zhu^2^

^1^Institute of Ecological Science, Guangzhou Key Laboratory of Subtropical Biodiversity and Biomonitoring, Guangdong Provincial Key Laboratory of Biotechnology for Plant Development, School of Life Sciences, South China Normal University, Guangzhou 510631, PR China

^2^Key Lab of Urban Environment and Health, Institute of Urban Environment, Chinese Academy of Sciences, Xiamen 361021, PR China

^3^School of Life Sciences, Sun Yat-sen University, Guangzhou 510275, PR China

^4^Guangdong Provincial Key Laboratory of Chemical Pollution, South China Normal University, Guangzhou 510006, PR China

^†^These authors contributed equally to this work.

^*^Corresponding author:

School of Life Sciences, South China Normal University, Guangzhou 510631, PR China

E-mail: Jin-tian Li ([lijintian@m.scnu.edu.cn](mailto:lijintian@m.scnu.edu.cn))

Tel.: +86 20 85211850; Fax: +86 20 85211850

**Number of supplementary figures**: 26

**Number of supplementary tables**: 17

**Supplementary figures**

**Figure S1. Summary of main results or findings of previous publications on microbial ARGs or ARs in mining-impacted environments. A.** A pie chart showing phylum-level taxonomic composition of bacterial strains that were isolated and tested for their tolerances to antibiotics. **B.** A pie chart showing the six most abundant ARGs in the six investigated mine sites. **C.** Pie charts showing the most abundant ARG type and their potential hosts in the two investigated mine sites. More details are provided in Table S1.

**Figure S2. Maps showing the locations of the mine sites in three datasets investigated in this study.** Detailed information on each studied mine site and the corresponding metagenomes are shown in Tables S2-S4.

**Figure S3. Comparison of Nonpareil curves of the selected metagenomes of the three sample types.** **A**. mine wastes. **B**. freshwater sediments. **C**. untreated urban sewage. The horizontal dashed lines indicate 100% and 95% taxonomy coverage. Each curve was generated from one metagenome. The empty circles indicate the data size and estimated average coverage of individual metagenomes, and the lines after the circles are projections of the fitted models.

**Figure S4. Compositions of ARG types on plasmids in the three datasets of this study.**

**Figure S5. Compositions of ARG resistance mechanisms in the three datasets of this study.**

**Figure S6. Abundant and ubiquitous ARG subtypes in the three datasets** **of this study. A-C.** Relative abundances of abundant ARG subtypes. Abundant ARG subtypes in a given dataset were defined as those with an average relative abundance > 1% in that dataset. Other types refer to ARG types beyond multidrug and bacitracin. **D-F.** Venn diagrams presenting the numbers of shared genes between abundant and ubiquitous ARGs. Ubiquitous ARGs in a given dataset were defined as those occurring in 100% of the samples in that dataset.

**Figure S7. Impacts of microbial taxonomic diversity (richness) on the diversity and abundance of MRGs in three sample types. A**. Pearson correlations between MRG richness and taxonomic richness. **B**. Pearson correlations between MRG abundance and taxonomic richness. Taxonomic richness was calculated on the species level. **C**. MRG abundance normalized by taxonomic richness.

**Figure S8.** **Percentages of microbial species carrying ARGs in the three sample types.** **A**. the overall pattern for all ARGs. **B-L**. patterns for dominant ARG types. ARG types beyond the top 10 most abundant types are grouped into “Others”, with an average relative abundance < 2%.

**Figure S9.** **Percentage of microbial species carrying MRGs in the three sample types. A**. the overall pattern for all MRGs. **B-L**. patterns for dominant ARG types. MRG types beyond the top 10 most abundant types are grouped into “Other metals”.

**Figure S10. Pearson correlations between *intI1* abundances and the total abundances of ARGs in the three datasets of this study.** All gene abundances are expressed as coverage normalized to data size (×/Gb). Correlations were calculated based on log-transformed abundances.

**Figure S11. Abundance, diversity and composition of MRGs in the studied mine sites.** Studied mine sites in each dataset are arranged on X-axis by latitude from south to north. Detailed information on each studied mine site and corresponding samples are shown in Tables S2-S4.

**Figure S12. Pearson correlations between total ARG abundance and concentration of zinc (Zn) or available manganese (Mn) in two datasets of this study.**

**Figure S13. Percentages of ARG-MRG carrying contigs in the total ARG-carrying contigs in the studied mine sites.**

**Figure S14. Co-occurrence patterns of ARGs and MRGs in the studied mine sites.** Analyses were based on high-quality metagenome-assembled genomes (MAGs: ≥ 95% completeness and ≤ 5% contamination) from two datasets of this study. Comparison of two groups was analyzed with Wilcoxon signed-rank test and comparison of multiple groups was analyzed with Kruskal-Wallis test. ns: non-significant; *: *p* < 0.05; **: *p* < 0.01; ***: *p* < 0.001.

**Figure S15. Compositions of nearest MRG types for each ARG type on plasmids in the** **three datasets** **of this study.**

**Figure S16. Numbers of ARGs per kb on chromosomes and plasmids in the three datasets of this study.** Comparison of chromosome and plasmid was analyzed with Wilcoxon signed-rank test. ***: *p* < 0.001.

**Figure S17. Percentages of ARG-MGE carrying plasmids in the total ARG-carrying plasmids in the studied mine sites.**

**Figure S18. Pearson correlations between the total abundance of ARGs and those of genes encoding integrase, resolvase and recombinase in the three datasets of this study.**

**Figure S19. Variations of ARG compositions explained by environmental factors in the China-T and SChina-S datasets.** ARG composition was analyzed at the subtype level. Statistical significance for each part of variation explained was checked using ANOVA test with 999 permutations. *: *p* < 0.05; ***: *p* < 0.001.

**Figure S20. Relative abundances of the top 10 dominant ARG-host phyla in the studies mine sites of two datasets of this study.** Results were based on analysis of high-quality MAGs.

**Figure S21. Diversity of hosts of each ARG type in two datasets of this study.** Results were based on analysis of high-quality MAGs. Stars (*) indicate ARG types which were classified as “Others” in the main text.

**Figure S22. Compositions of ARG types in the top 20 ARG-host families and the top 50 ARG-host genera in two datasets of this study.** Results were based on analysis of high-quality MAGs.

**Figure S23. Relative abundances of the high-quality ARG-carrying MAGs that can be classified into species level in the studied mine sites in two datasets of this study.** Potential pathogens are labeled red.

**Figure S24. Numbers of virulence factor genes in the 54 potential pathogenic MAGs and 30 non-pathogenic MAGs. A.** Number of ORFs annotated to virulence factor (VF) genes on individual MAGs. **B-E.** Comparison between the pathogenic and non-pathogenic MAGs by average number of ORFs annotated to non-secreted VFs, secreted VFs, non-secreted toxin genes, and secreted toxin genes. Error bars indicate standard deviation. *p*-values are derived from Wilcoxon signed-rank test.

**Figure S25. Comparisons of the numbers of ARGs, multidrug ARGs, MRGs, and multimetal MRGs between the 54 potential pathogenic MAGs and 30 non-pathogenic MAGs.** *p*-values are derived from Wilcoxon signed-rank test.

**Figure S26. Pearson correlations between the total abundances of ARGs and those of MRGs after removing the ORFs annotated as both ARG and MRG in the three datasets of this study.**

**Supplementary tables**

**Table S1. Summary of previous publications on microbial antibiotic resistance genes (ARGs) or antibiotic resistances (ARs) in mining-impacted environments.**

**Table S2. Detailed information of the mine sites and their corresponding metagenomes (samples) in the global public AMD-related dataset.**

**Table S3. Detailed information of the mine sites and their corresponding metagenomes (samples) in the China mine tailings dataset.**

**Table S4. Detailed information of the mine sites and their corresponding metagenomes (samples) in the South China AMD sediment dataset.**

**Table S5. The self-constructed database of microbial genes encoding mobile genetic elements (MGEs).**

**Table S6. The self-constructed database of pathogenic microbial species.**

**Table S7. Information of 30 randomly picked non-pathogenic MAGs and the 54 potential pathogenic MAGs.**

**Table S8. Sub-sampling of 30 randomly selected mine waste metagenomes** **newly generated in this study.**

**Table S9. Detailed information of 30 randomly selected public untreated sewage metagenomes.**

**Table S10. Detailed information of 30 randomly selected public freshwater sediment metagenomes.**

**Table S11. Detailed information of abundant ARG subtypes and ubiquitous ARG subtypes in the studied mine sites.**

**Table S12. Good-quality ARG-carrying MAGs (completeness** ≥ **75% and contamination** ≤ **10%) recovered from the China mine tailings dataset.**

**Table S13. Good-quality ARG-carrying MAGs recovered from the South China AMD sediment dataset.**

**Table S14. Summary of good-quality ARG-carrying MAGs from the China mine tailings dataset and the South China AMD sediment dataset.**

**Table S15. Genes annotated as both ARG and MRG in the studied mine sites.**

**Table S16. Average relative abundances of the genes annotated as both ARG and MRG in the studied mine sites.**

**Table S17. Core non-regulatory multidrug ARGs possessed by the pathogenic microbial species that can be matched to specific high-quality MAGs in this study.**

**Figure S1**

**
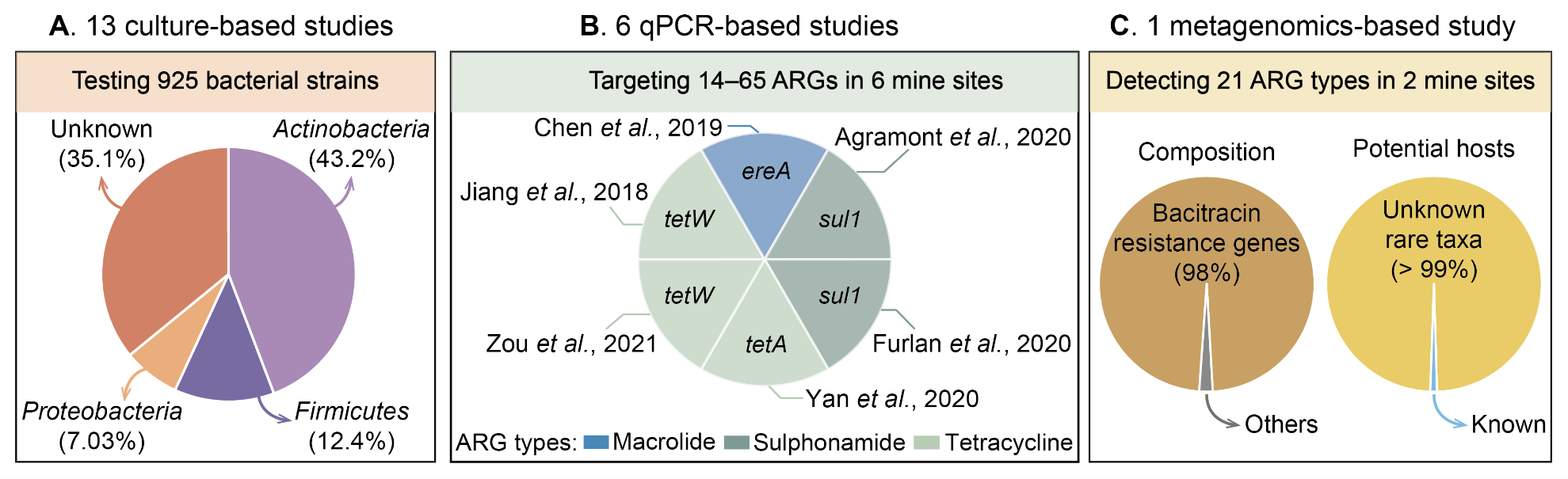
**

**Figure S2**

**
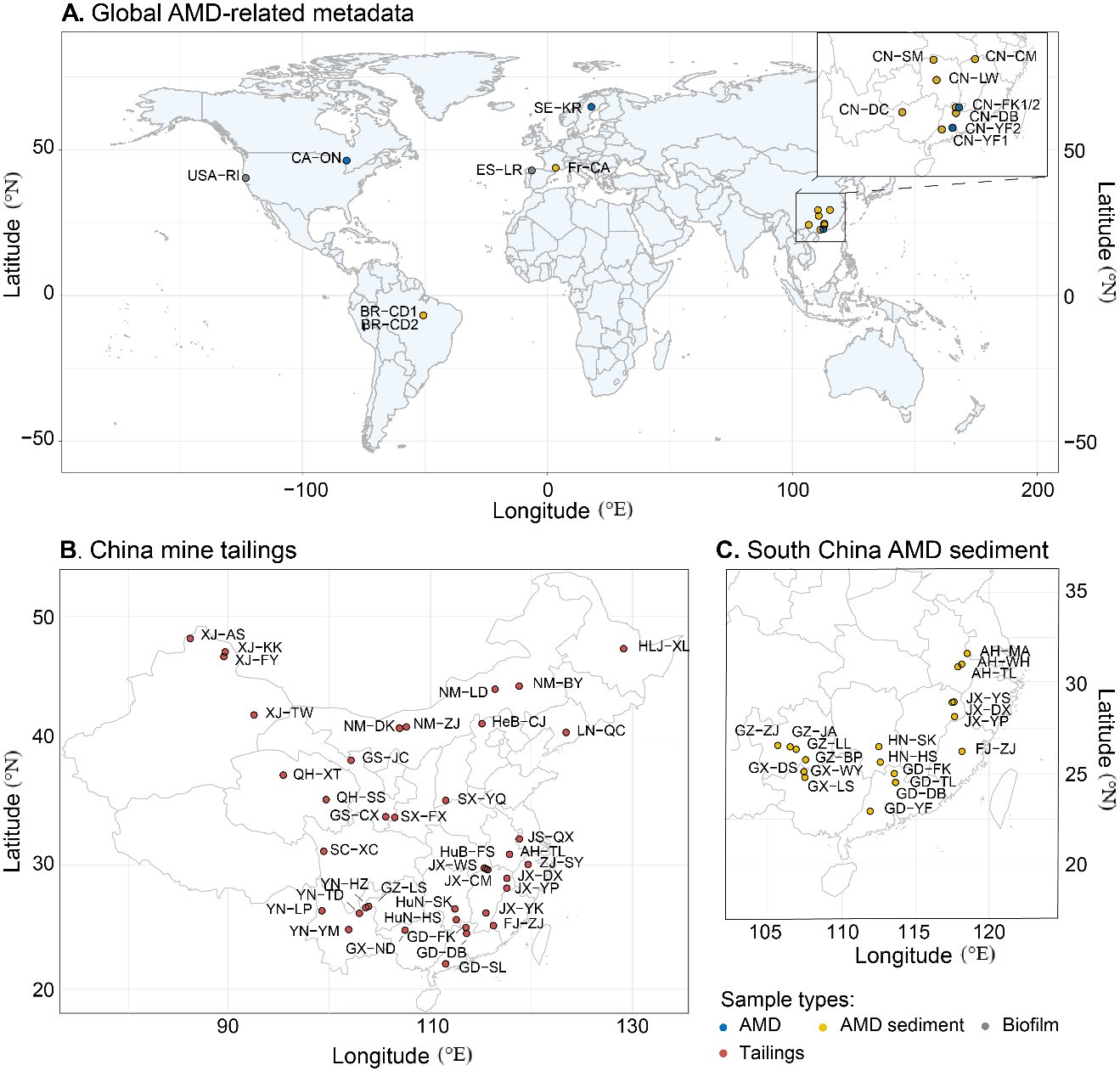
**

**Figure S3**

**
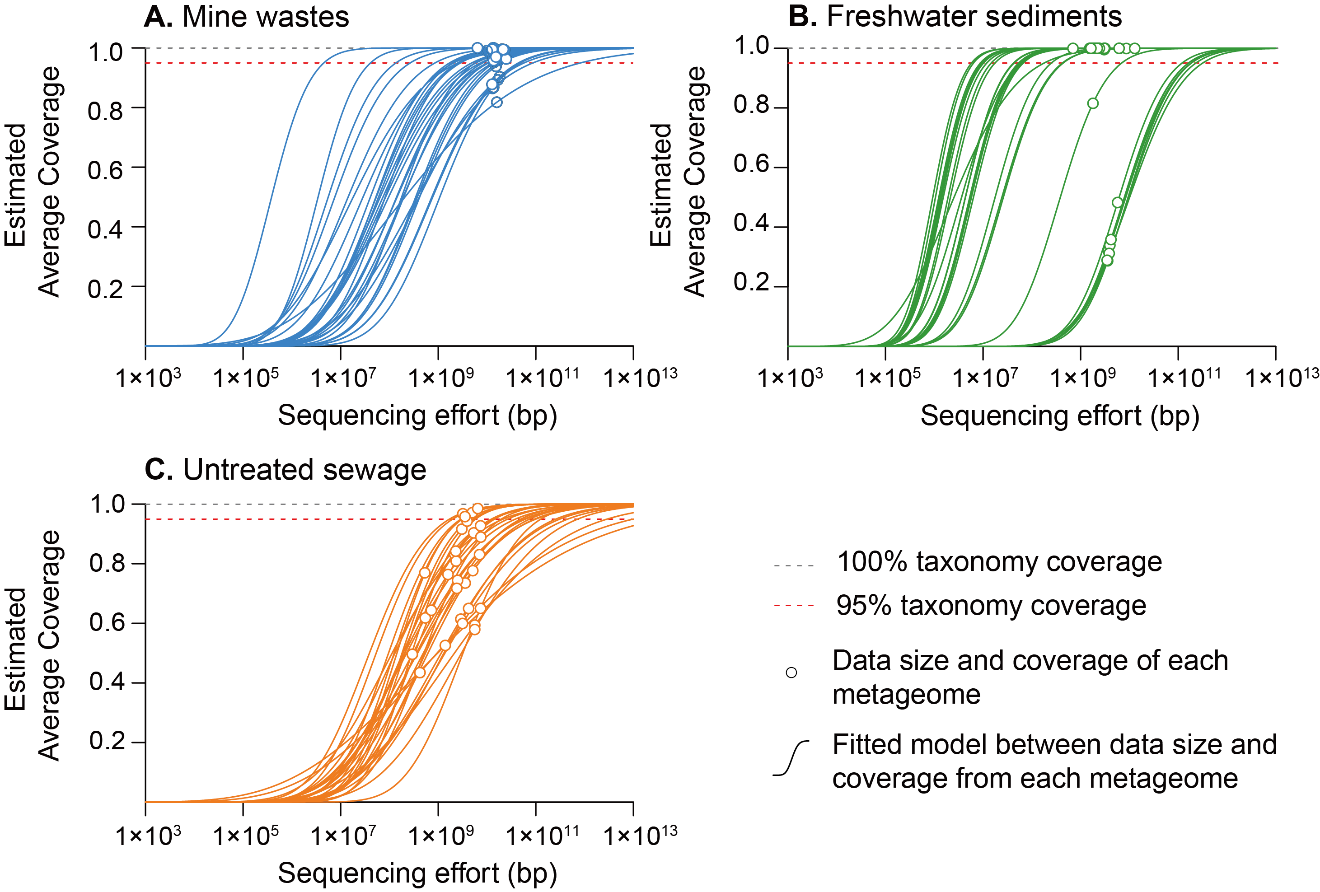
**

**
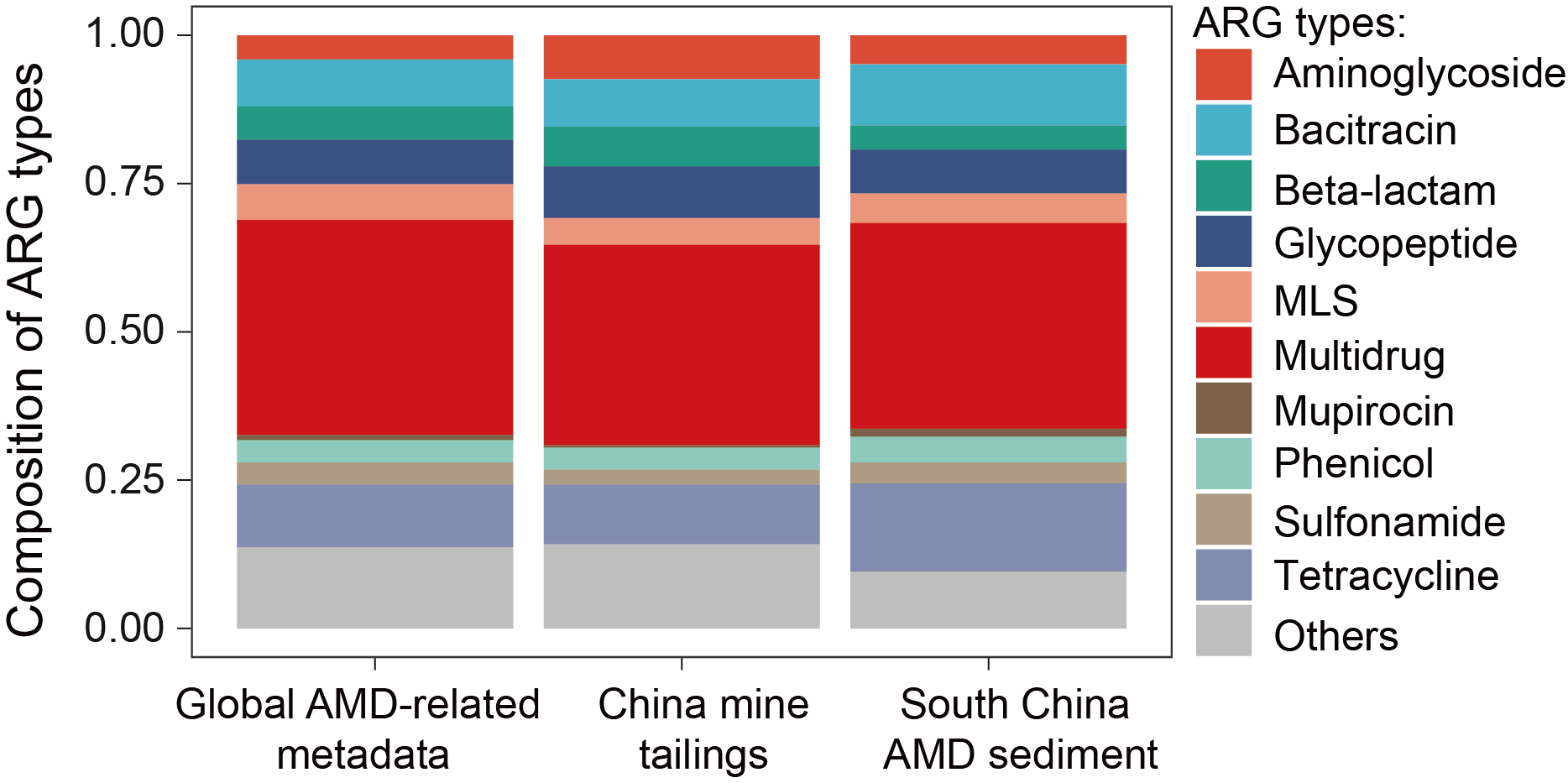
Figure S4**

**Figure S5**


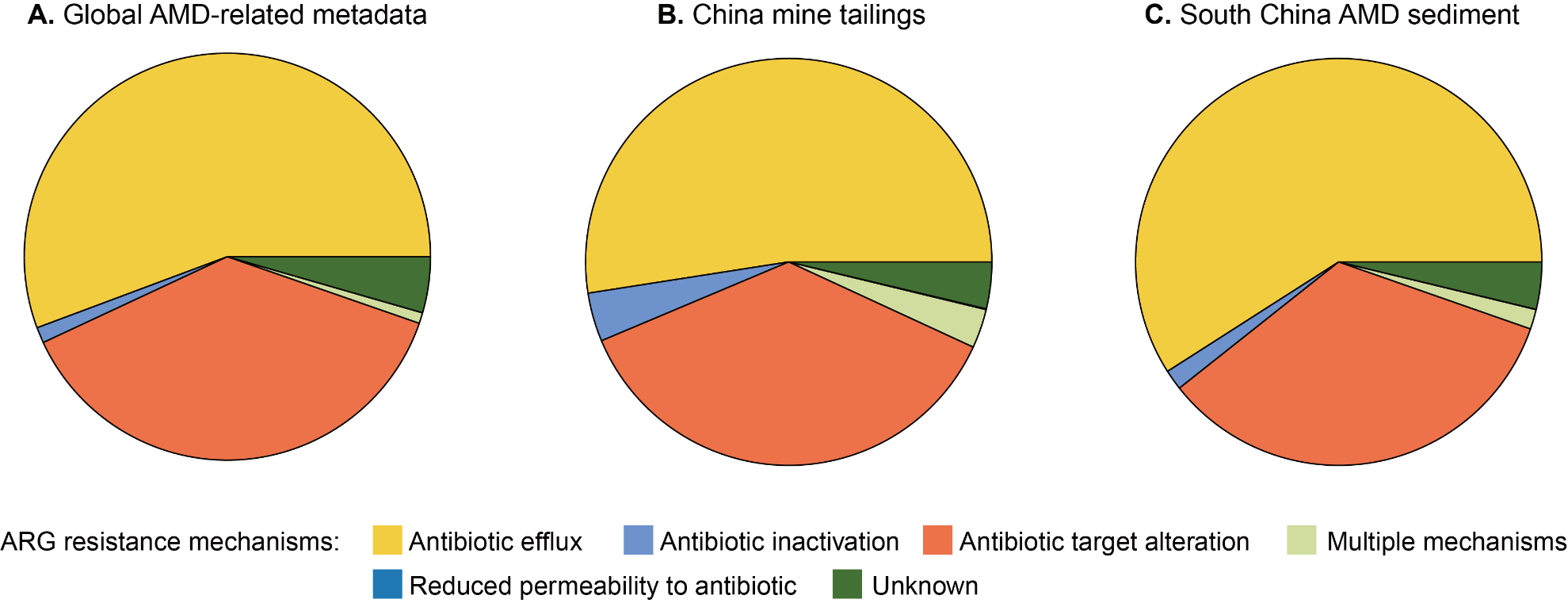


**
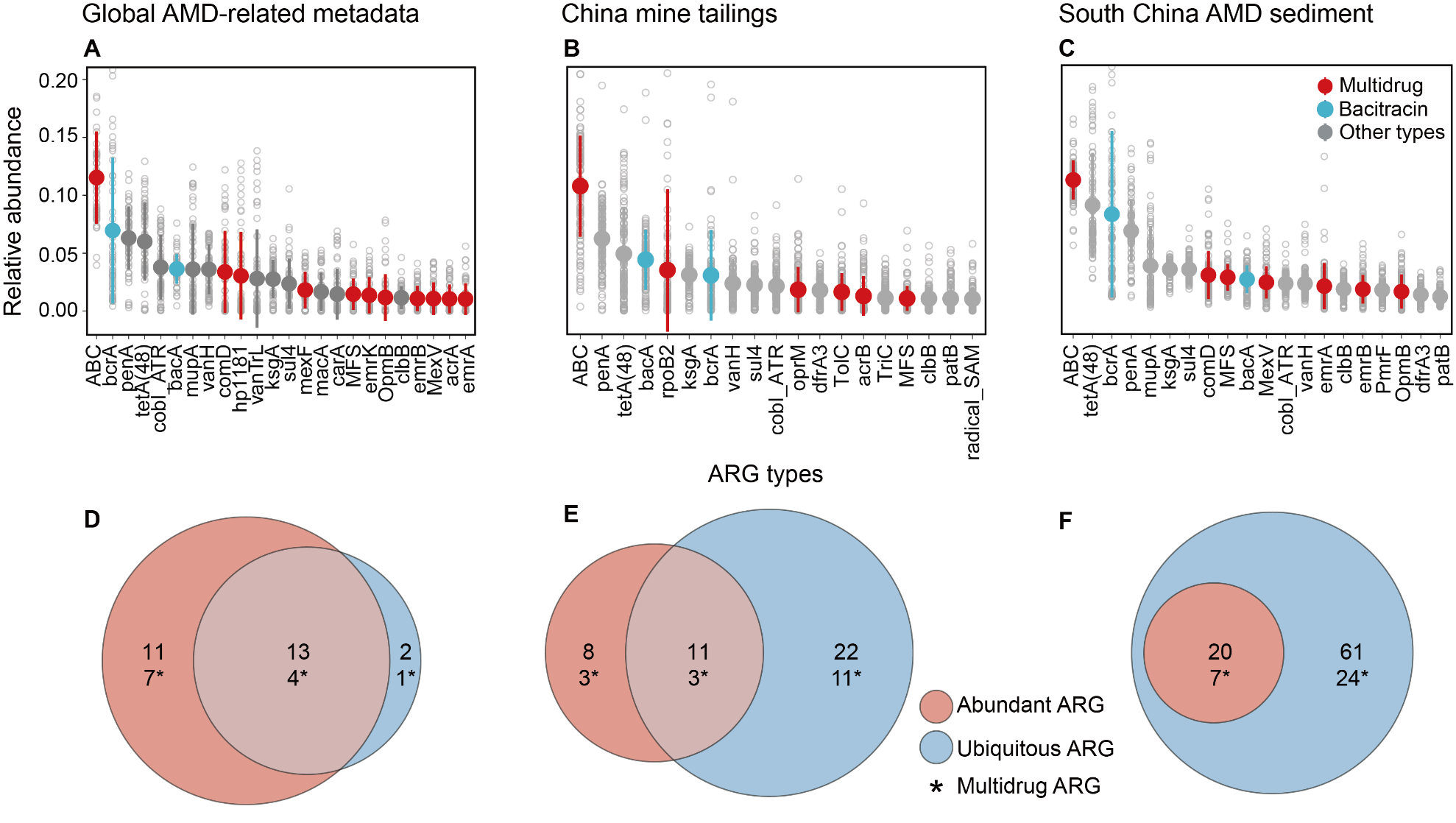
Figure S6**

**Figure S7**

**
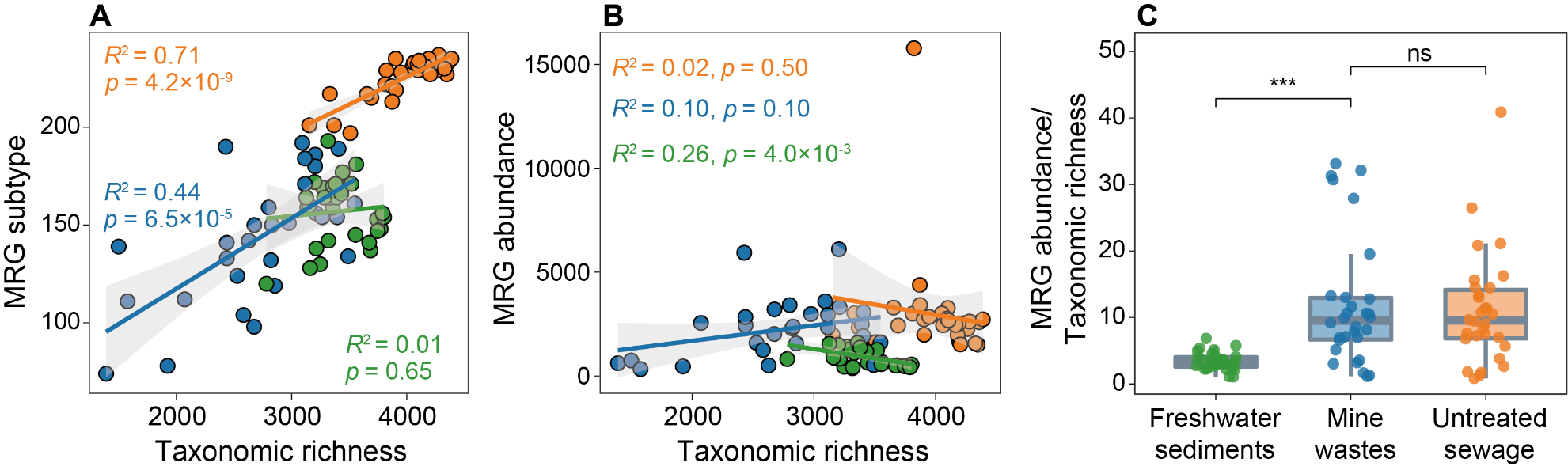
**

**Figure S8**

**
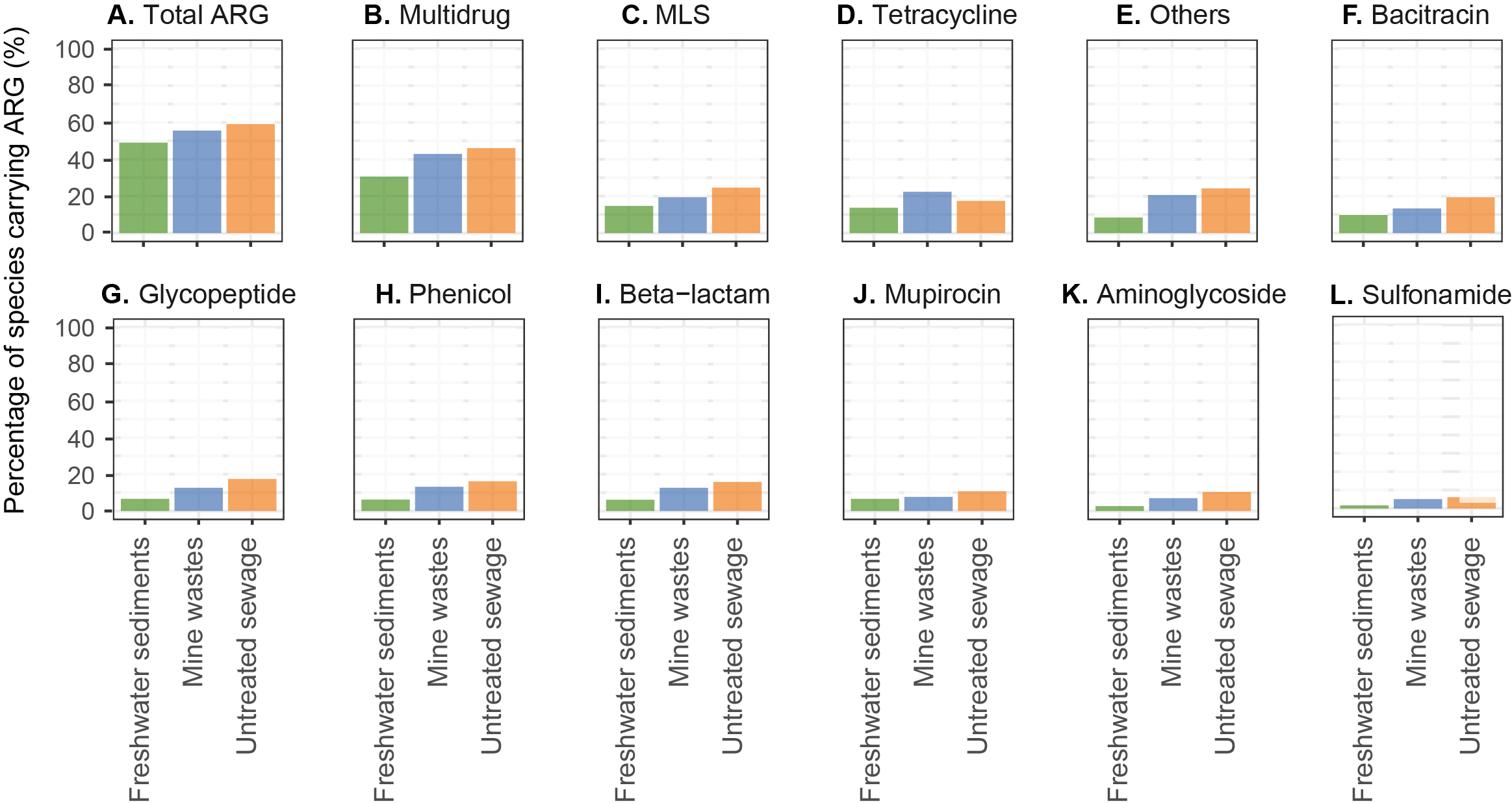
**

**Figure S9**

**
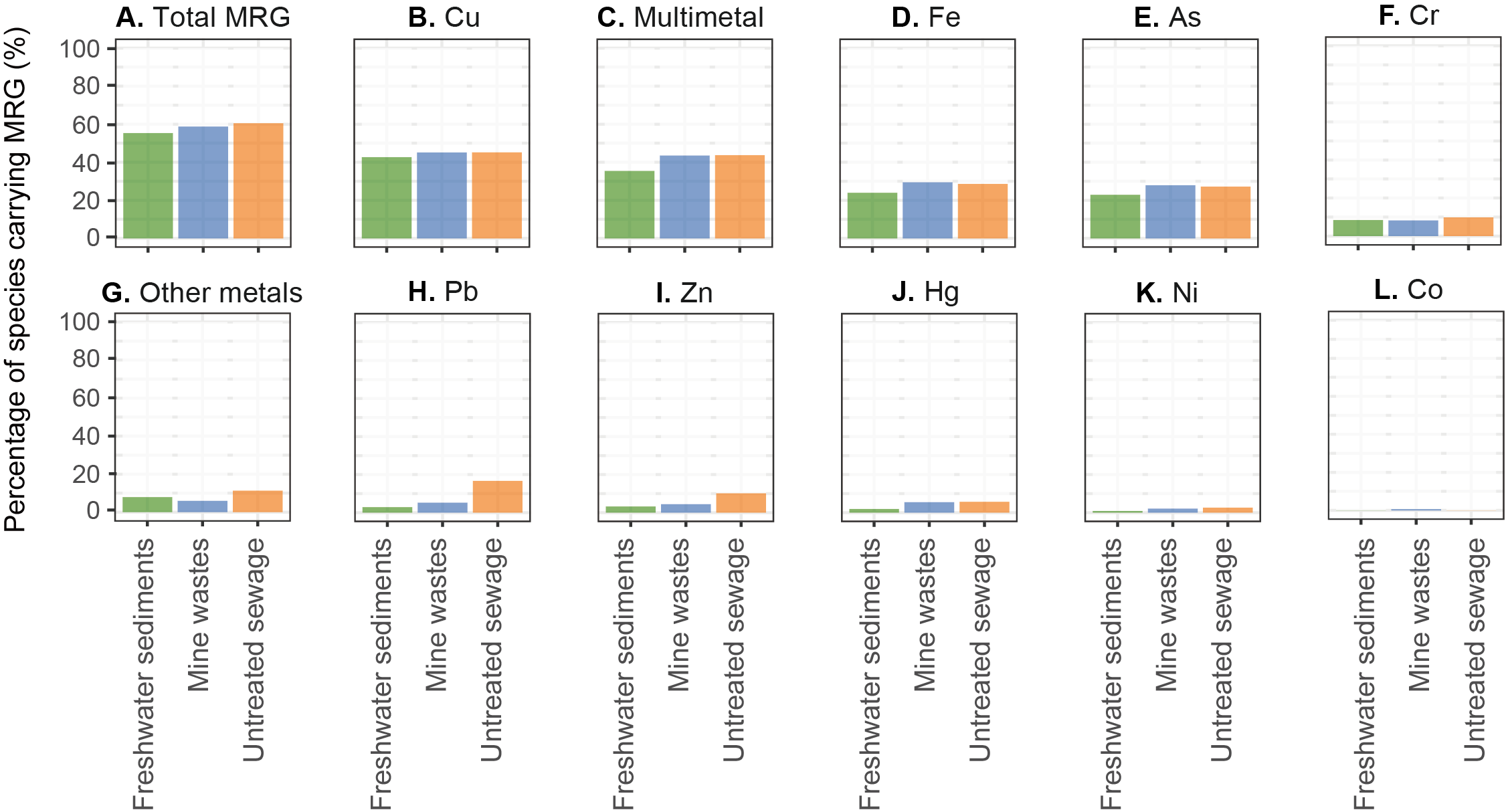
**

**
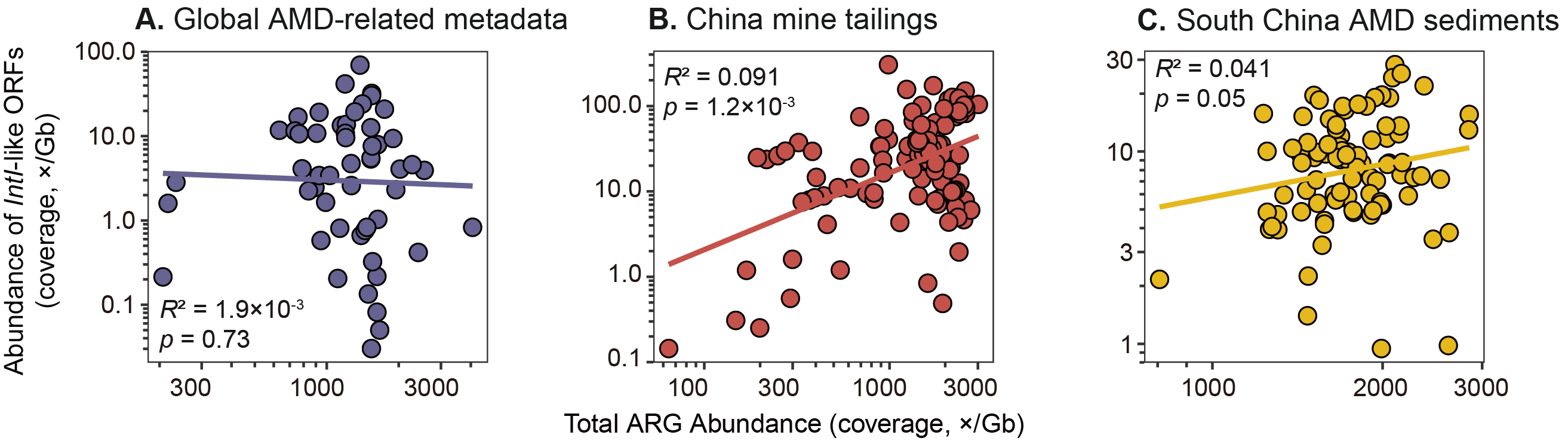
Figure S10**

**Figure S11**

**
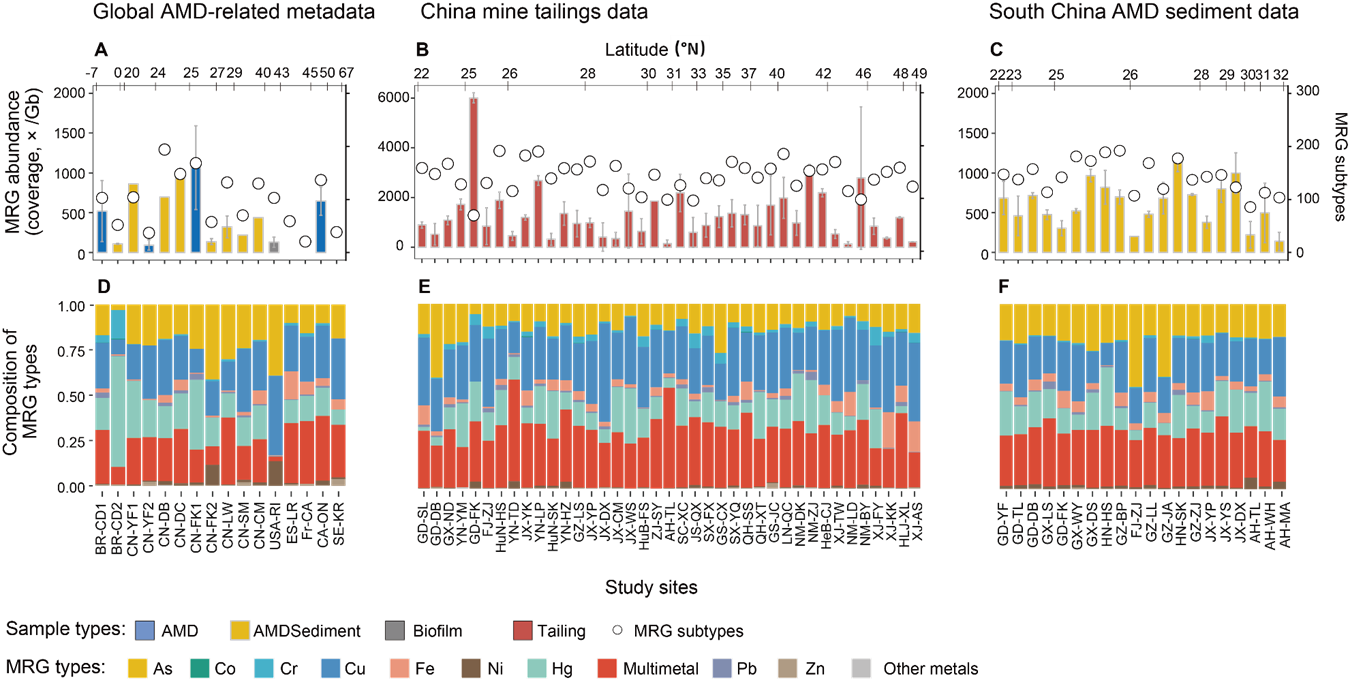
**

**Figure S12**

**
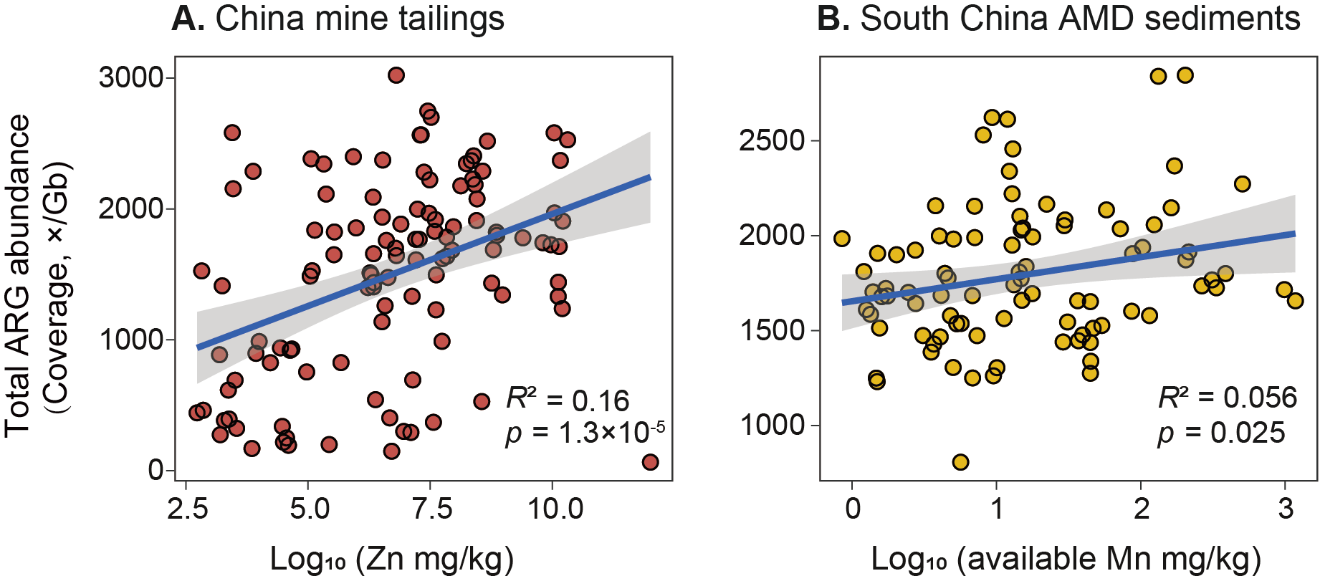
**

**Figure S13**


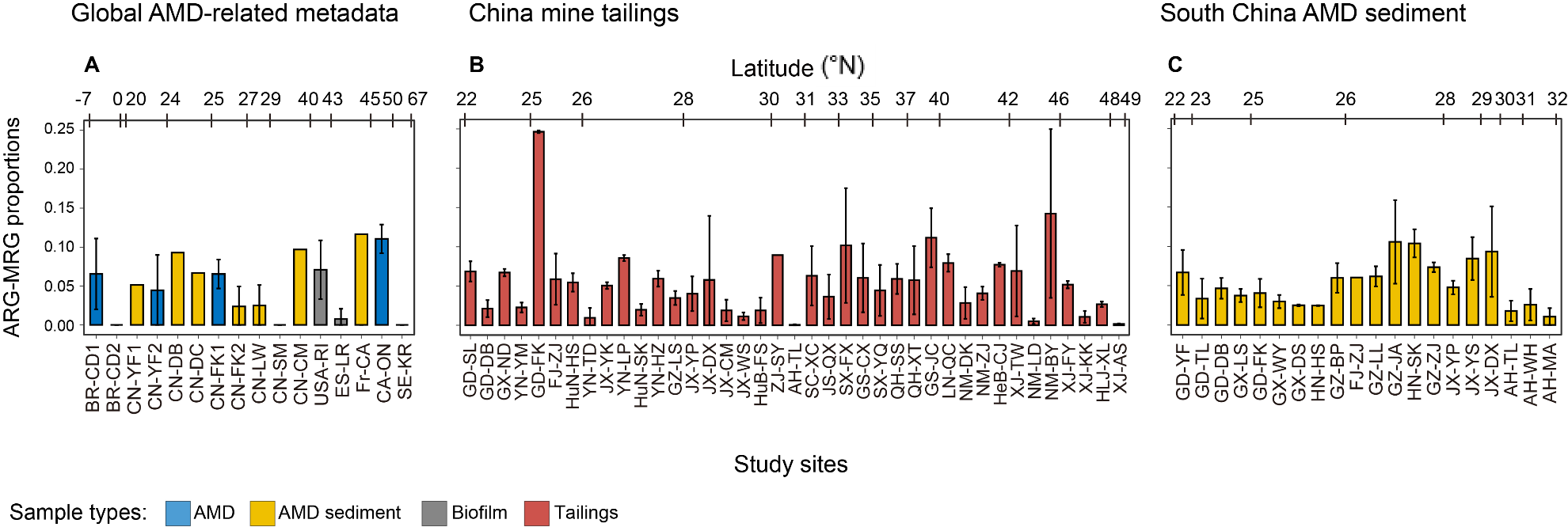


**Figure S14**

**
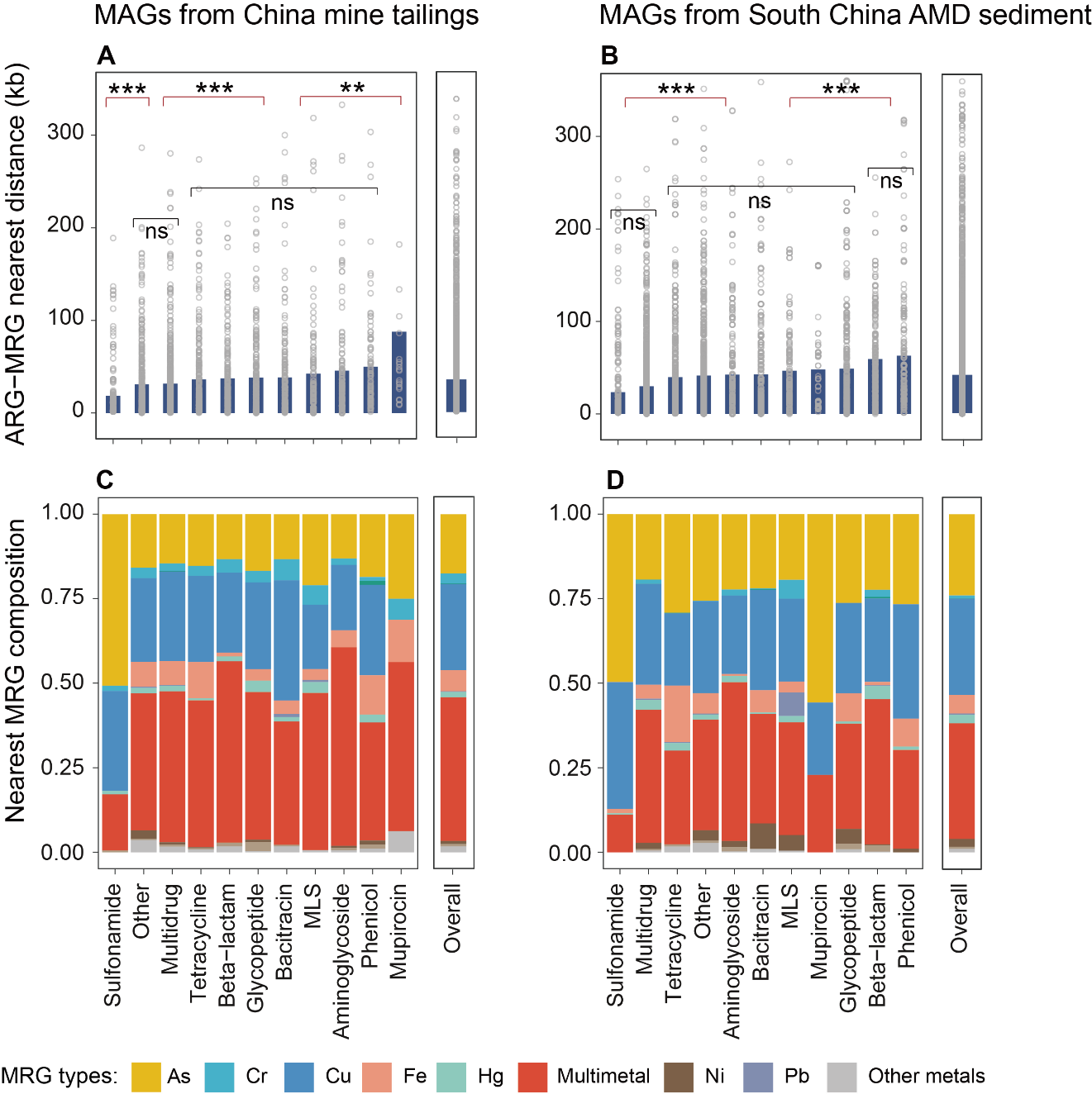
**

**
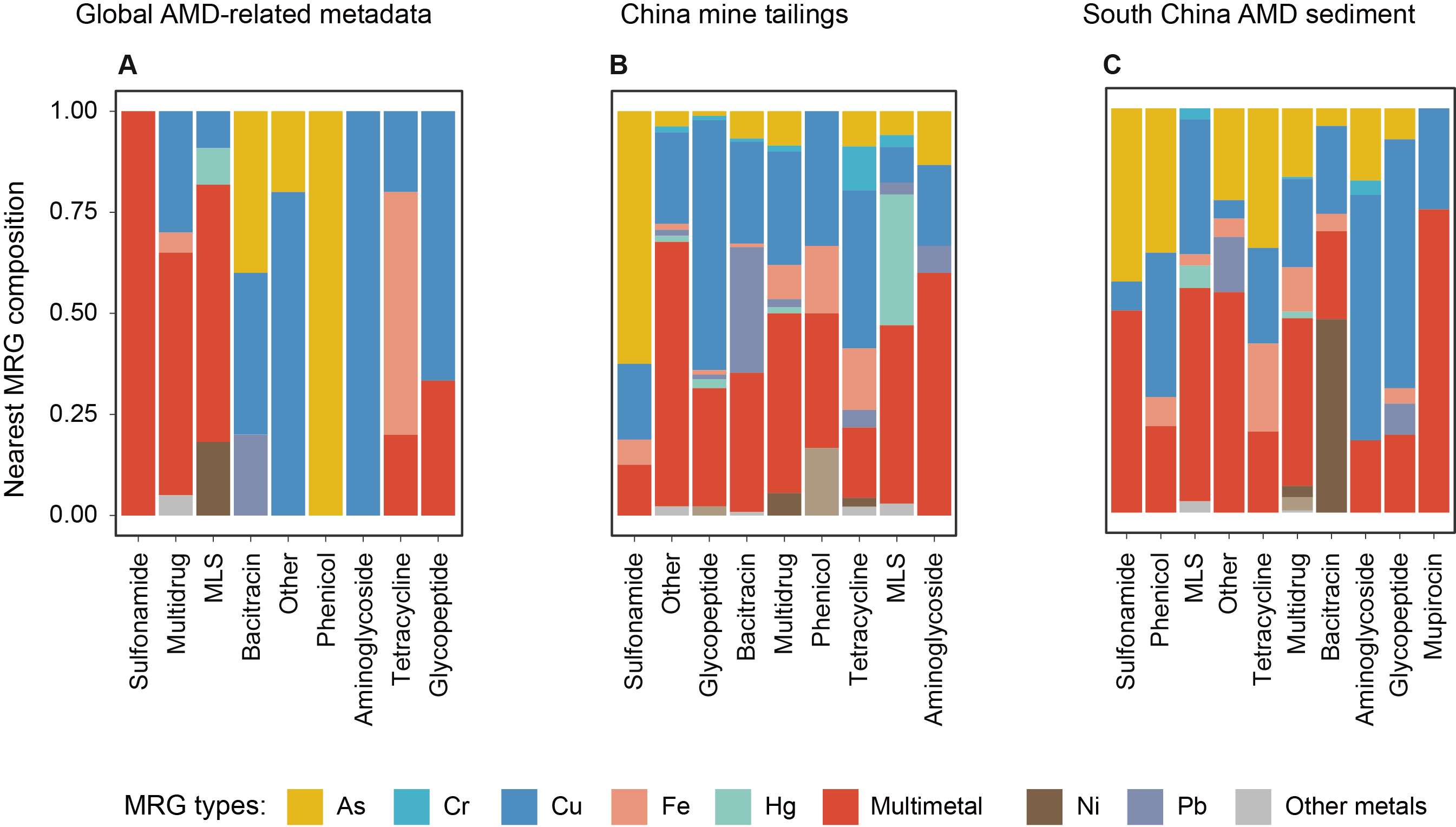
Figure S15**

**Figure S16**


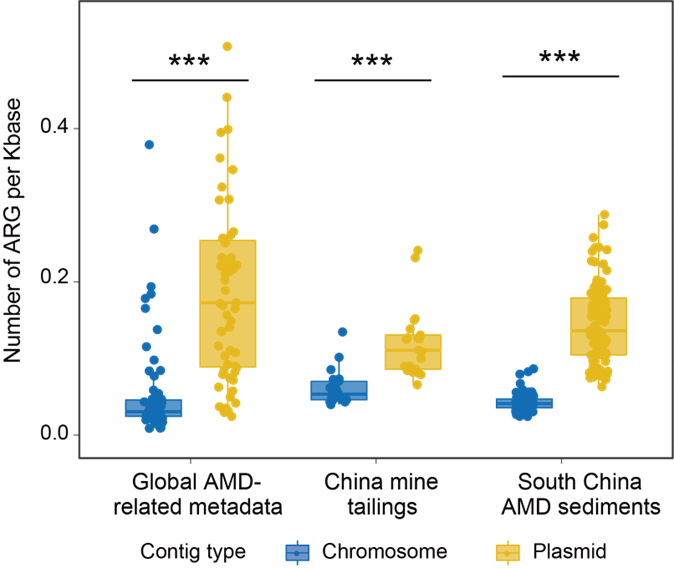


**Figure S17**

**
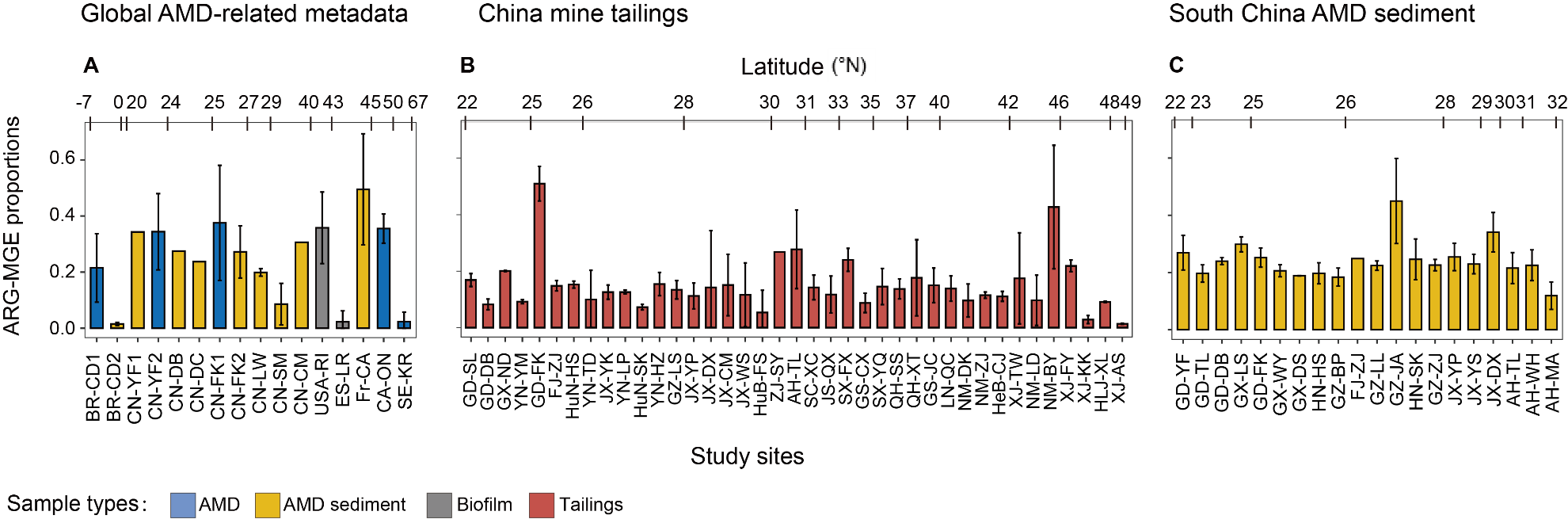
**

**Figure S18**

**
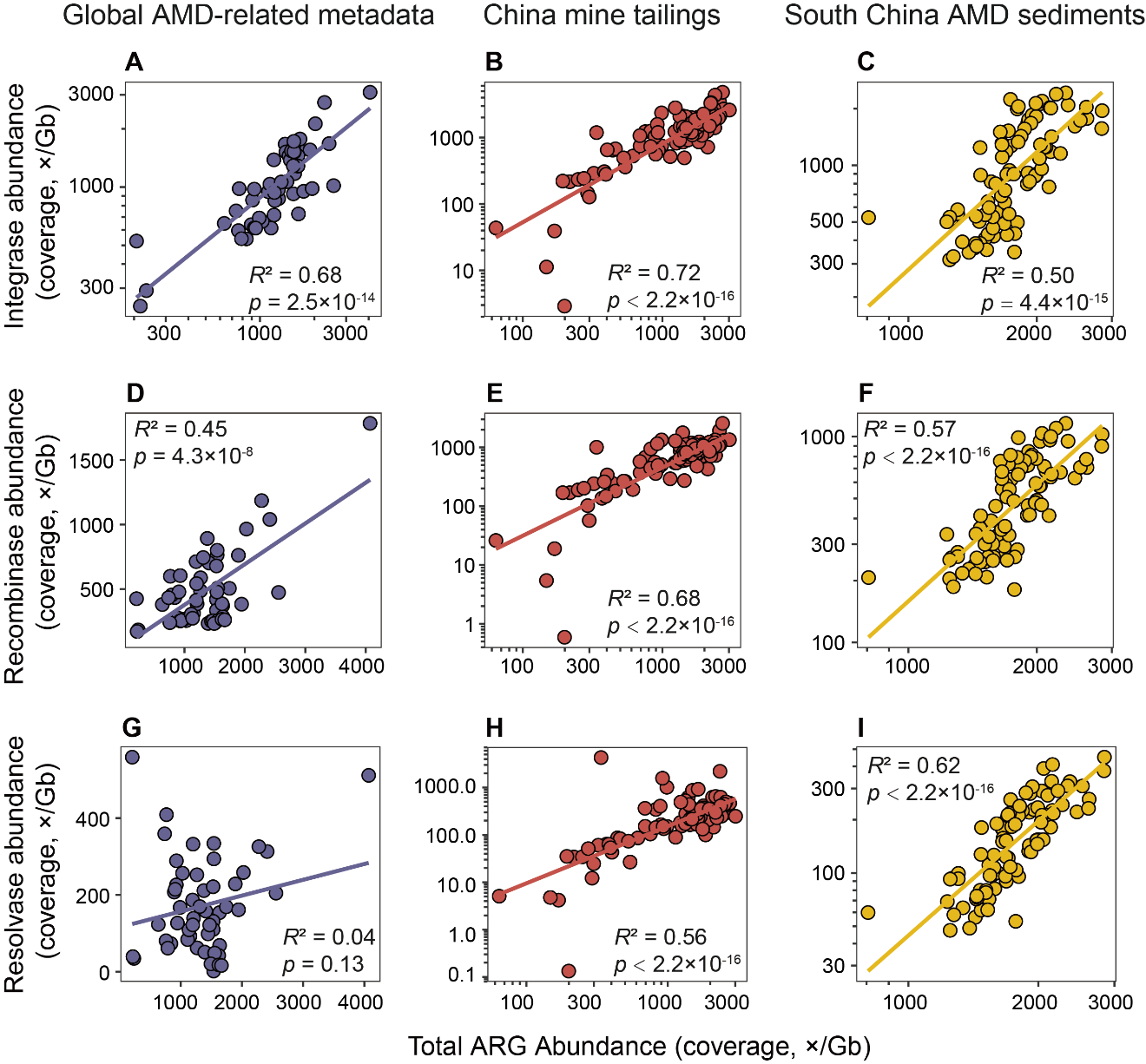
**

**Figure S19**

**
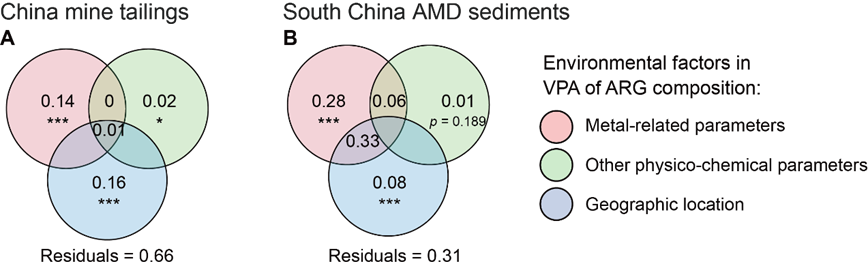
**

**Figure S20**

**
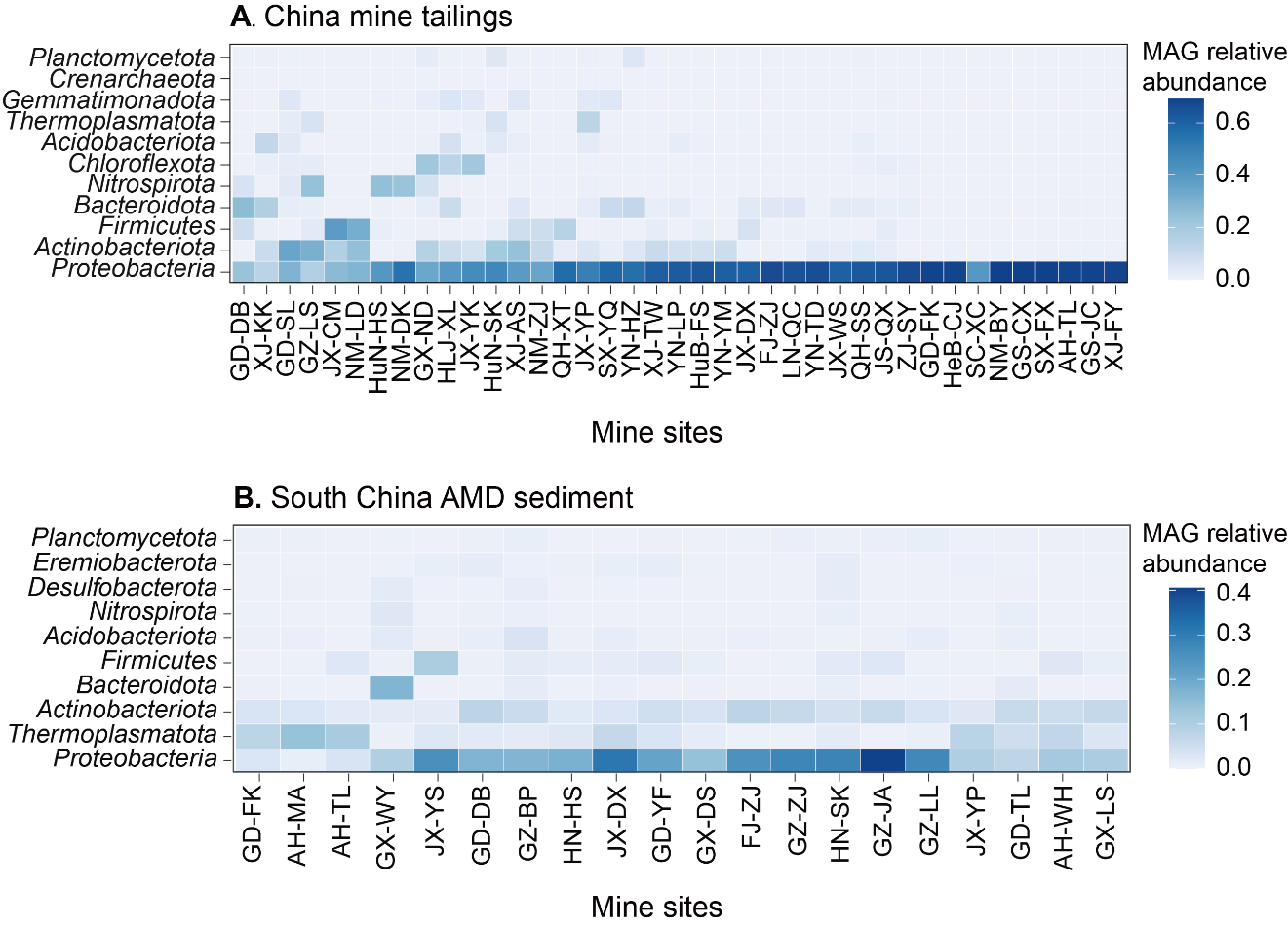
**

**Figure S21**


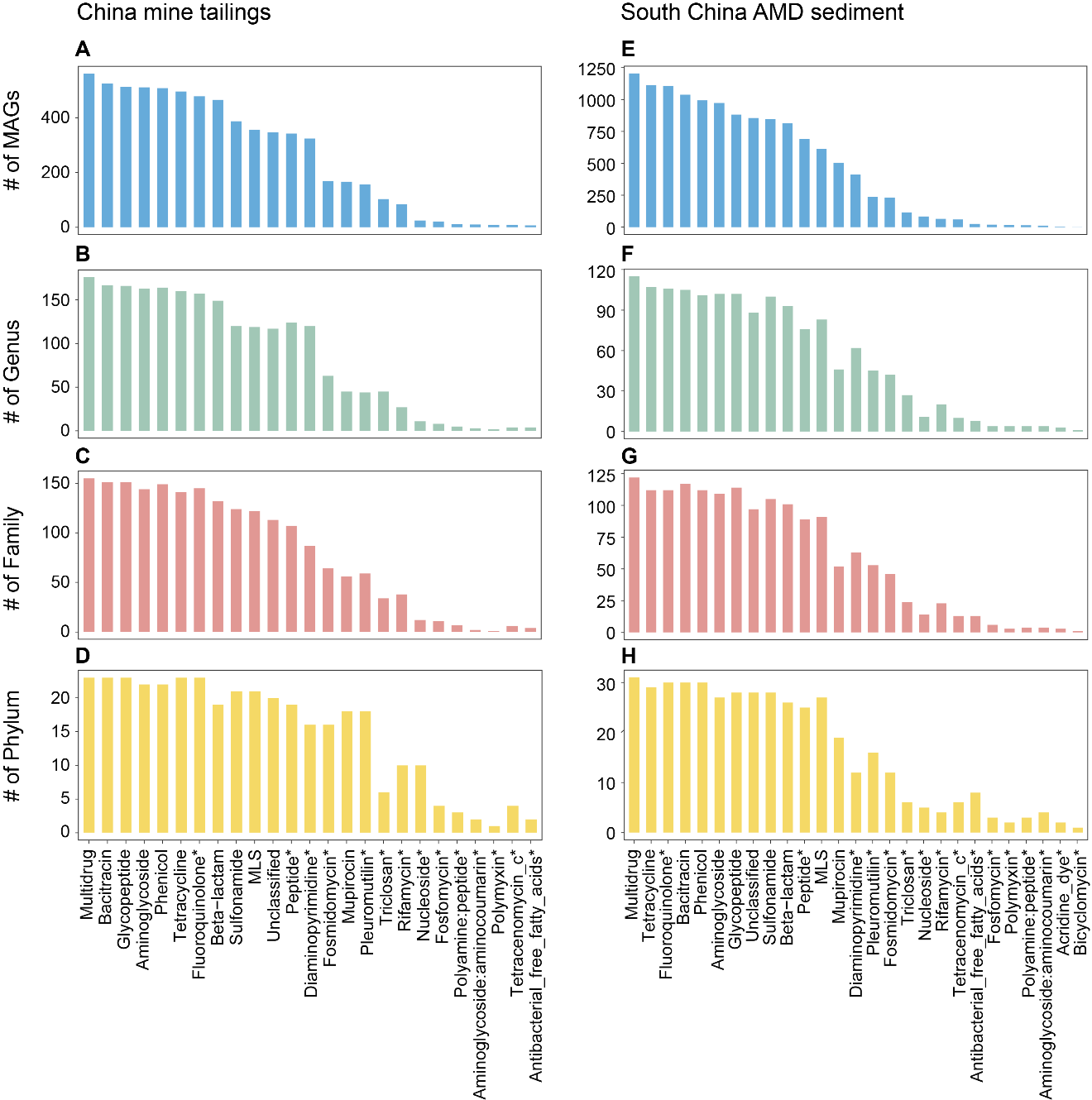


**Figure S22**


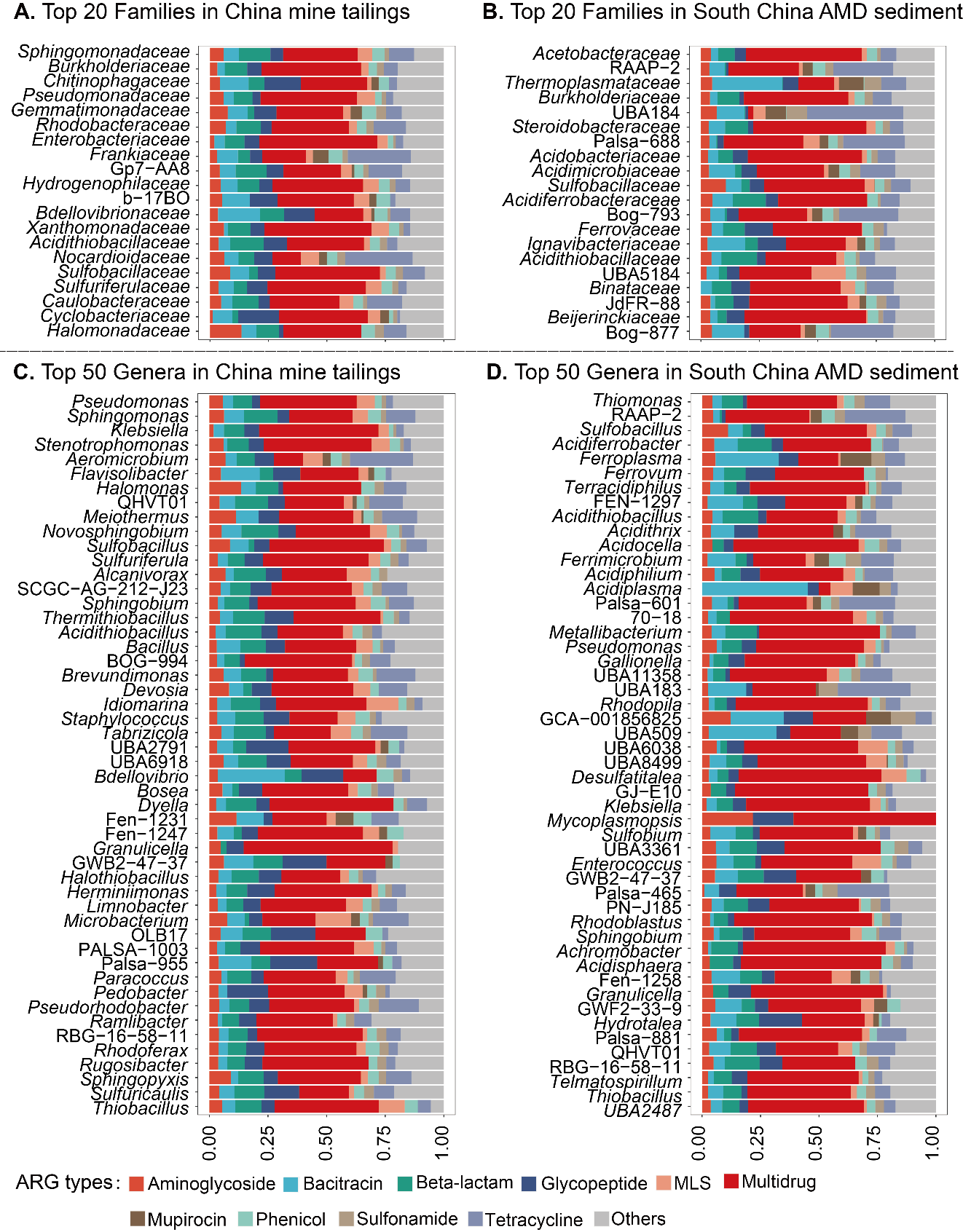


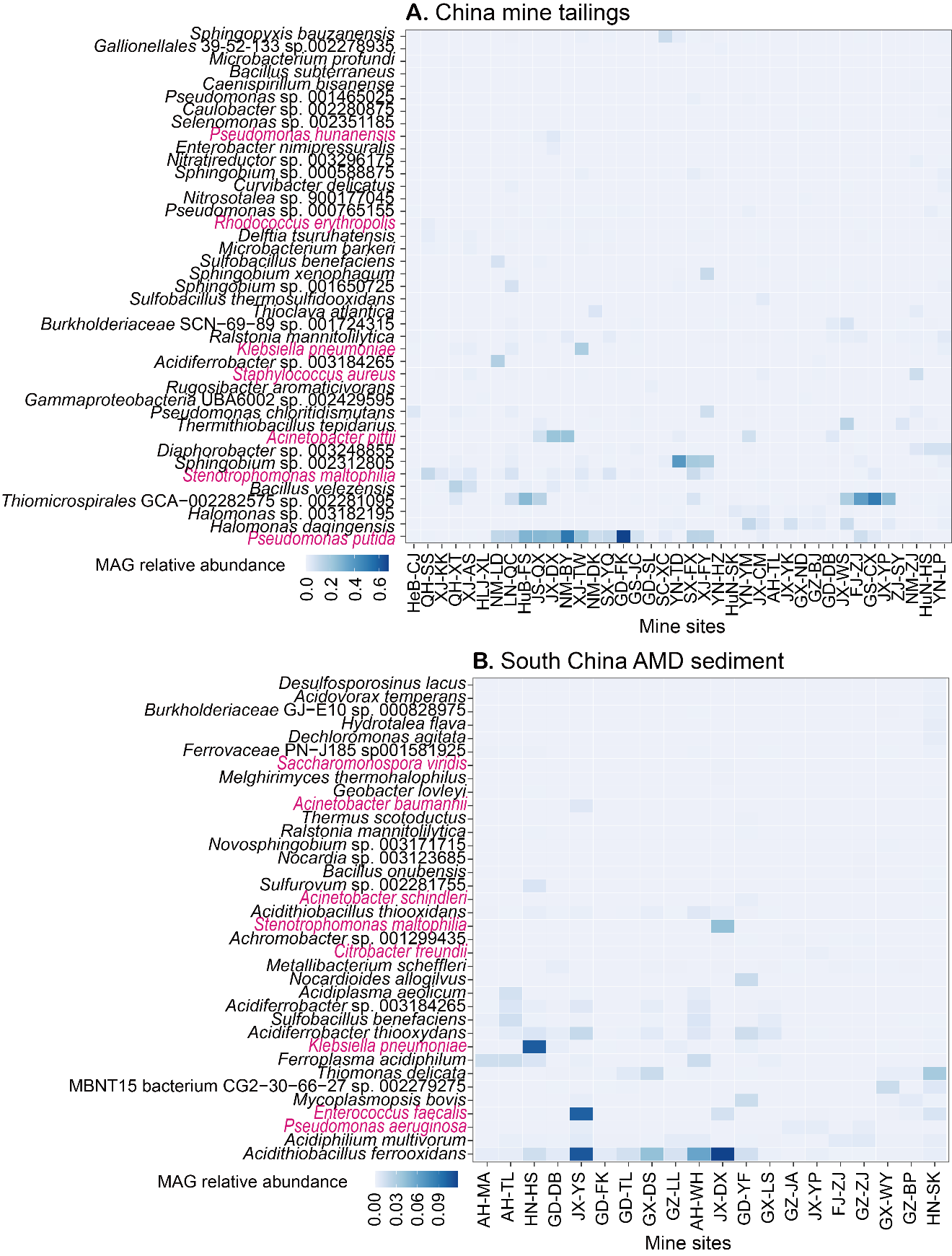
**Figure S23**

**Figure S24**


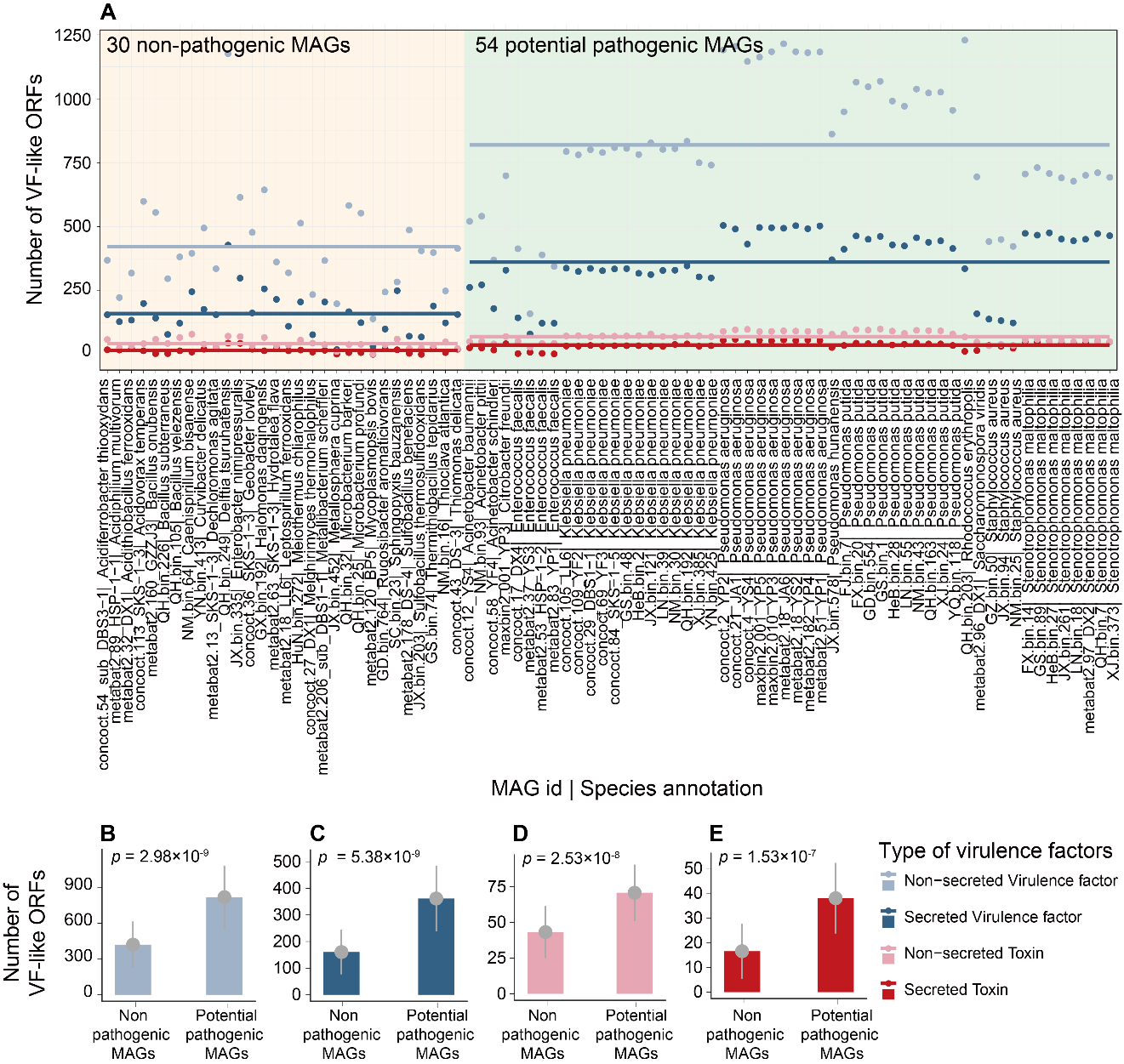


**Figure S25**


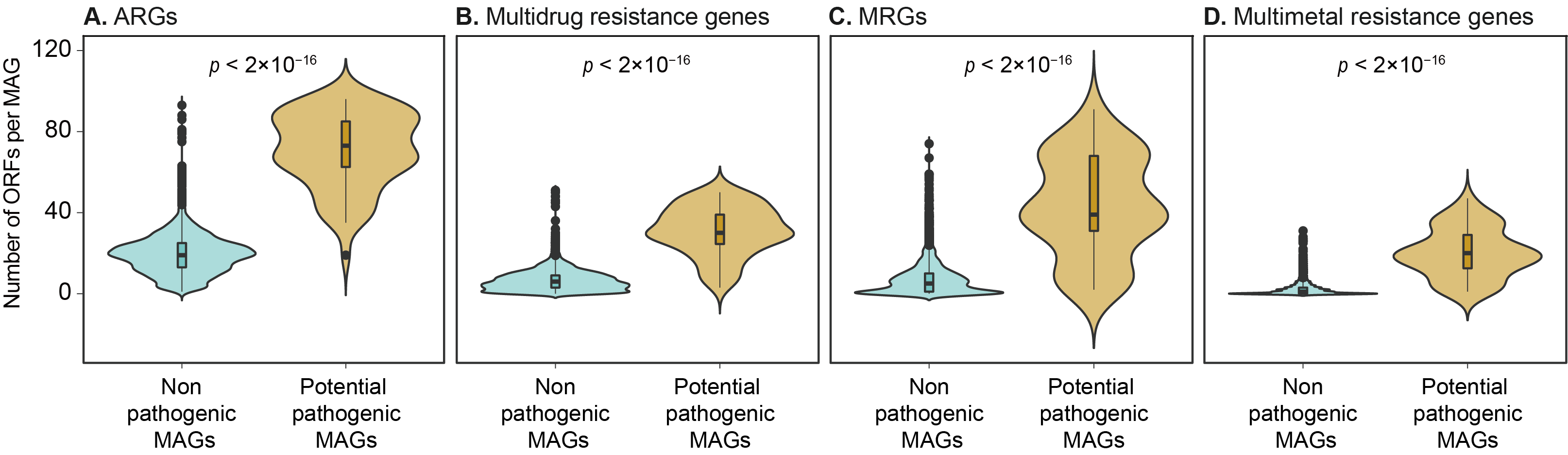


**Figure S26**


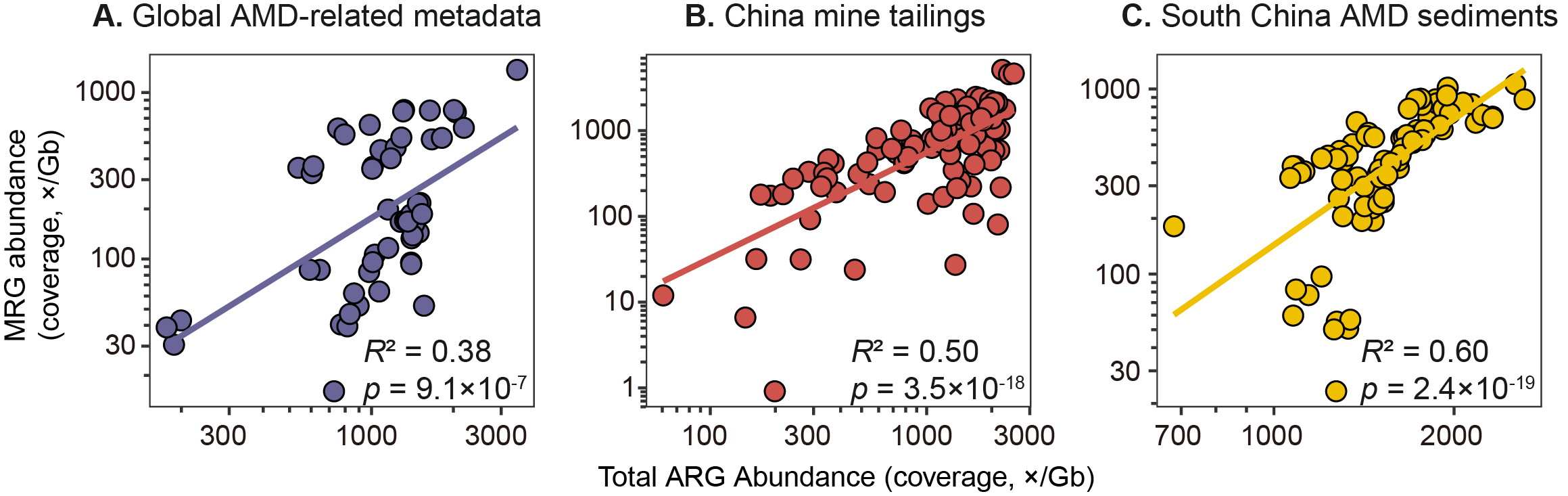

Supplement: Supplementary file 1 — Supplementary figures [file 41396_2022_1258_MOESM1_ESM.docx]
